# TRIM31 facilitates K27-linked polyubiquitination of SYK to regulate antifungal immunity

**DOI:** 10.1038/s41392-021-00711-3

**Published:** 2021-08-06

**Authors:** Xueer Wang, Honghai Zhang, Zhugui Shao, Wanxin Zhuang, Chao Sui, Feng Liu, Xiaorong Chen, Jinxiu Hou, Lingli Kong, Hansen Liu, Yi Zheng, Bingyu Liu, Tian Chen, Lei Zhang, Xinming Jia, Chengjiang Gao

**Affiliations:** 1 Shandong Provincial Key Laboratory of Infection and Immunity & Department of Immunology, School of Biomedical Sciences, Shandong University, Jinan, Shandong China; 2 Department of Pathogenic Biology, School of Biomedical Sciences, Shandong University, Jinan, Shandong China; 3 Clinical Medicine Scientific and Technical Innovation Center, Shanghai Tenth People’s Hospital, Tongji University School of Medicine, Shanghai, China

**Keywords:** Infectious diseases, Innate immunity

## Abstract

Spleen tyrosine kinase (SYK) is a non-receptor tyrosine kinase, which plays an essential role in both innate and adaptive immunity. However, the key molecular mechanisms that regulate SYK activity are poorly understood. Here we identified the E3 ligase TRIM31 as a crucial regulator of SYK activation. We found that TRIM31 interacted with SYK and catalyzed K27-linked polyubiquitination at Lys375 and Lys517 of SYK. This K27-linked polyubiquitination of SYK promoted its plasma membrane translocation and binding with the C-type lectin receptors (CLRs), and also prevented the interaction with the phosphatase SHP-1. Therefore, deficiency of *Trim31* in bone marrow-derived dendritic cells (BMDCs) and macrophages (BMDMs) dampened SYK-mediated signaling and inhibited the secretion of proinflammatory cytokines and chemokines against the fungal pathogen *Candida albicans* infection. *Trim31*^−/−^ mice were also more sensitive to *C. albicans* systemic infection than *Trim31*^+/+^ mice and exhibited reduced Th1 and Th17 responses. Overall, our study uncovered the pivotal role of TRIM31-mediated K27-linked polyubiquitination on SYK activation and highlighted the significance of TRIM31 in anti-*C. albicans* immunity.

## Introduction

Fungal infections are major public health threats, especially HIV patients,^1^ immune-compromised people with hematopoietic stem cell transplantation (HSCT) and chemotherapy,^2^ or primary immune deficiencies.^3^ Recent studies also found that lots of fungal species bloom in the gut of COVID-19 patients.^4^ Many species of fungi are responsible for invasive fungal infections (IFIs), and about 1.5 million people die from IFI yearly.^5^ *Candida albicans* are among the most prevalent opportunistic fungal pathogens of humans. Although several anti-fungal drugs are effective, the mortality rate of *Candida* infections exceeds 40%.^6^ Current antifungal drugs for IFI treatment are limited and may cause undesirable side effects. Moreover, the rapid emergence of drug resistance is a growing problem.^7,8^ Therefore, a better understanding of how the host immune system counteracts fungal infections is vital for the development of novel therapeutic strategies to combat candidiasis.

During fungal infection, CLRs and the Toll-like receptor (TLR) play essential roles in host defense against fungal pathogens by recognizing various fungal surface components.^9–11^ The CLRs are mainly expressed on neutrophils, macrophages, and dendritic cells such as Dectin-1, Dectin-2, Dectin-3, and Mincle, and they recognize β-glucan, α-mannan, and glycolipids of fungi, respectively.^12–15^ Upon recognition of respective ligands, CLRs initiate the phosphorylation of the Tyr-X-X-leu motif (termed ‘ITAM’) in the Dectin-1 cytoplasmic tail the adaptor FcRγ and recruitment of FcRγ to Dectin-2 or Mincle, which serves as a docking site for SYK. These events promote SYK translocation and activation,^16^ which then activates PLCγ2^17^and PKCδ.^18^ The central adaptor protein caspase recruitment domain-containing protein 9 (CARD9), B cell leukemia-lymphoma 10 (BCL10) and mucosa-associated lymphoid tissue 1 (MALT1) then form the CARD9–BCL10–MALT1 (CBM) complex to activate mitogen-activated protein (MAP) kinase, along with NF-κB and NFAT transcription factors,^19^ leading to the production of proinflammatory cytokines and chemokines.^20^ Moreover, these cytokines and chemokines promote neutrophil infiltration, macrophage maturation, and T cell differentiation.^21,22^ The Th1 and Th17 subsets of T helper cells have been demonstrated to participate in host defense against fungal infection via IFN-γ, IL-17A, and IL-17F production, which further activate and recruit macrophages and neutrophils.^23^

SYK is a non-receptor tyrosine kinase that contains two Src homology 2 (SH2) domains and a carboxy-terminal kinase domain. Further, there are two domains (termed interdomains A and B) located, respectively, between the two SH2 domains and between the kinase domain and the second SH2 domain. SYK exists in an autoinhibited structure due to the combination of interdomain A and interdomain B with the kinase domain, which blocks the substrates of access to the catalytic domain in resting cells. As an essential adaptor and kinase of the CLR signaling complex proximal to the plasma membrane, SYK activity must be fine-tuned during fungal infection. Previous study suggested that the activation of this autoinhibited confirmation may be determined by interaction partners or post-translational modifications rather than by intramolecular interactions.^24^ For instance, ZAP70, one of the SYK tyrosine kinase family, is ubiquitinated by NRDP1, which dephosphorylates Zap70 and terminates TCR signaling through recruiting the phosphatase-like proteins STS1 and STS2.^25^ It has also been reported that the ubiquitination of SYK plays a critical role in innate antifungal immunity. SYK can be catalyzed for Lys48 (K48)-linked polyubiquitination by E3 ubiquitin ligase CBL-B, leading to the degradation of phosphorylated SYK.^26–28^ However, whether SYK activation is being modified by other E3 ubiquitin ligases besides CBL-B is completely unknown.

Members of the tripartite motif (TRIM) family proteins are well known as E3 ubiquitin ligases which contain a conserved RING-finger domain, one or two B-boxes, and a coiled-coil domain.^29^ The TRIM family participates in various biological processes including fighting against HIV and tumor progression by modulating the K48- or K63-linked polyubiquitination to the targets. Several members, including TRIM10,^30^ TRIM15,^31^ TRIM26,^32^ TRIM27,^33^ TRIM31,^34^ TRIM38,^35^ TRIM39,^36^ and TRIM40,^37^ are shown to be involved in innate immune responses, possibly due to the encoding genes locating in the locus which encoding the major histocompatibility complex (MHC) class I proteins.^38^ Our laboratory has previously reported that TRIM31 is involved in RIG-I-like receptors (RLR) signaling during RNA virus infection. TRIM31 promotes the catalyzation of K63-linked polyubiquitination of mitochondrial antiviral signaling protein (MAVS), and this modification facilitates MAVS aggregation which further induces Interferon-β (IFN-β) production to inhibit virus replication.^34^ We also found that TRIM31 can promote proteasomal degradation of NLR Family Pyrin Domain Containing 3 (NLRP3) and therefore inhibits NLRP3 inflammasome activation and attenuates the DSS-induced colitis inflammation.^39^ Until now, the role of the TRIM family during fungal infection had rarely been reported.

In this study, we screened several important members of the TRIM family to investigate the possible roles of this family in regulating SYK activity during fungal infections. We identified that TRIM31 is an essential positive regulator for SYK. Particularly, TRIM31 catalyzed the K27-linked polyubiquitination at Lys375 and Lys517 of SYK, which subsequently promoted SYK translocation and binding to CLRs, and decreased the association of SYK with the phosphatase SHP-1. This therefore up-regulated SYK kinase phosphorylation and activity. Consequently, TRIM31 protected hosts from the lethal systemic infection with *C. albicans* in the mouse model by increasing pro-inflammatory cytokines and chemokines production, which aided in clearing the pathogen more efficiently. Overall, our study first reported that SYK was modified through K27-linked polyubiquitination by TRIM31 upon fungal infection, and also uncovered its significance in positively regulating the activation of SYK-associated signaling cascades and the resultant anti-fungal immunity.

## Results

### TRIM31 interacts with and promotes polyubiquitination of SYK

**Fig. 1:**
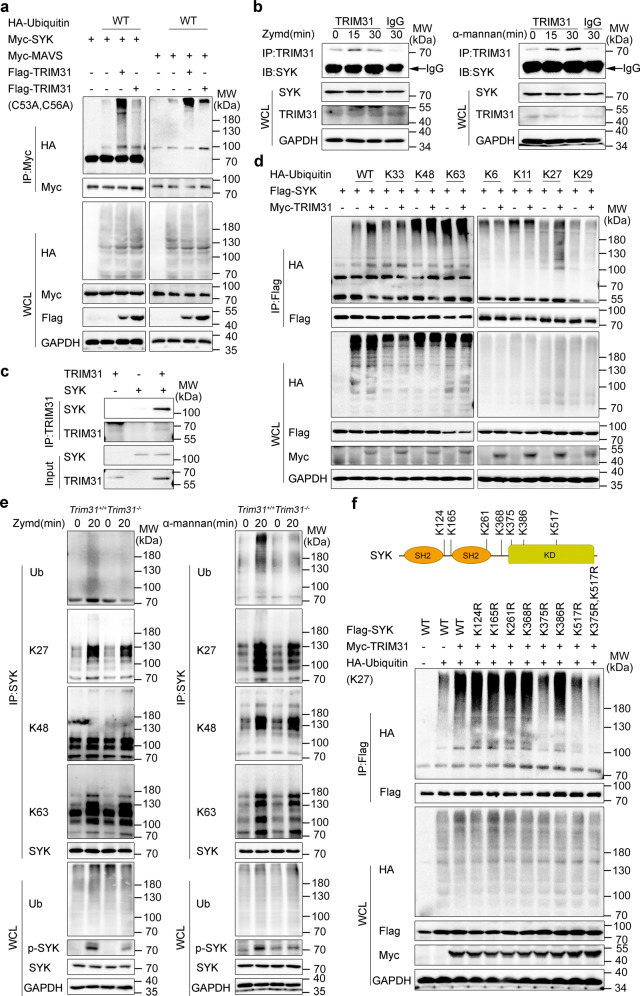
TRIM31 catalyzes the K27-linked polyubiquitination of SYK. **a** HEK293T cells were transfected with Myc-SYK or Myc-MAVS, HA-ubiquitin (WT), Flag-TRIM31, or Flag-TRIM31 (C53A, C56A) and ubiquitination assays were performed. **b** Co-IP analysis of the endogenous interaction between TRIM31 and SYK in BMDCs stimulated with Zymd (left) or α-mannan (right) for 0-30 mins. **c** In vitro Co-IP analysis of TRIM31 with SYK using recombinant TRIM31 and SYK. **d** ubiquitination assays analysis of SYK in HEK293T cells transfected with Flag-SYK, Myc-TRIM31, and HA-Ubiquitin or its mutants (K6, K11, K27, K29, K33, K48, and K63). **e** Immunoprecipitation analysis of ubiquitination of endogenous SYK in *Trim31*^+/+^ and *Trim31*^−/−^ BMDCs stimulated with Zymd (left) and α-mannan (right) for indicated time points. **f** Schematic diagram of lysine residues in SYK for ubiquitination identified by mass spectrometry (top). Immunoprecipitation analysis of ubiquitination of SYK and its mutants in HEK293T cells transfected with Myc-TRIM31, Flag-SYK (WT or mutants) along with HA-Ubiquitin (K27) (bottom). Antibodies (left margins). The above experiments were repeated at least twice with similar results, and the representative data were shown (**a–f**)

SYK is an important proximal kinase for downstream signaling from various cell surface receptors, such as CLRs, Fc receptors, and complement receptors. It has been reported that phosphorylation is vital in inducing SYK activation.^16^ However, whether SYK is being modified by ubiquitination and the role of SYK ubiquitination are rarely reported. To fill up this knowledge gap, we set out to screen which E3 ubiquitin ligase(s) may catalyze SYK ubiquitination. We chose several TRIM family members which have been demonstrated to perform essential roles in innate antiviral immunity including TRIM14,^40^ TRIM25,^41^ TRIM30α,^42^ TRIM41,^43^ TRIM56,^44^ TRIM62,^45^ TRIM65,^46^ and eight TRIM members which were encoded within the MHC-I region. We over-expressed these TRIM family proteins as well as SYK in HEK293T cells. It was found that TRIM25, TRIM31, and TRIM39 increased SYK polyubiquitination (Supplementary Fig. S1a). Notably, TRIM31 had the highest efficiency to catalyze the ubiquitination of SYK (Supplementary Fig. S1a). Thus, we focused on TRIM31 in this study. To further confirm that TRIM31 is involved in SYK ubiquitination, HEK293T cells were co-transfected with Myc-SYK, HA-ubiquitin, and Flag-TRIM31 WT or TRIM31 (C53A, C56A) which lacks its enzymatic activity. We found that the polyubiquitination of SYK was readily detected in the presence of WT TRIM31. While, TRIM31 (C53A, C56A) failed to do so (Fig. 1a). As a positive control, TRIM31 ubiquitinated MAVS as previously reported in our lab (Fig. 1a).^34^ SYK is a key adaptor of anti-fungal innate immune response via CLRs. To test whether TRIM31 catalyzes the polyubiquitination of other molecules in the CLR signaling pathway, the molecules in this upstream signaling pathway including CARD9, BCL10, PLCγ2, SYK, SHP-2, and PKCδ were also studied. Interestingly, we found that TRIM31 specifically catalyzed SYK polyubiquitination, but not other CLR upstream signaling proteins (Supplementary Fig. S1b, c). Furthermore, TRIM31 did not ubiquitinate the CLRs including Dectin-1, Dectin-2, Dectin-3, Mincle, and the adaptor FcR-γ (Supplementary Fig. S1d). Overall, these results indicate that TRIM31 catalyzes the polyubiquitination of SYK.

Given that TRIM31 catalyzes the polyubiquitination of SYK, we proposed that TRIM31 might interact with SYK. We over-expressed TRIM31-GFP and other molecules in the CLR signaling pathway and found that TRIM31 interacted with SYK, but not with other molecules in HEK293T cells (Supplementary Fig. S2a, b). Further, the interaction between endogenous TRIM31 and SYK was increased in BMDCs upon stimulation with Zymd or α-mannan (Fig. 1b). We also confirmed the direct interaction between TRIM31 and SYK in vitro using recombinant proteins (Fig. 1c). Confocal microscopy results showed that TRIM31 colocalized with SYK (Supplementary Fig. S2c). SYK contains N terminal two tandem SH2 domains and a carboxy-terminal kinase domain. To investigate which domain in SYK was necessarily targeted by TRIM31, we constructed three deletion mutants of SYK and evaluated their ability to interact with TRIM31. We found that the kinase domain of SYK was necessary for the interaction with TRIM31 (Supplementary Fig. S2d).

We next investigated which type of SYK polyubiquitination that were mediated by TRIM31. Then we over-expressed HA-ubiquitin mutants K6, K11, K27, K29, K33, K48, and K63, which contain the one lysine residue at the indicated position, and other lysine residues were substituted by arginine. As expected, TRIM31 catalyzed the SYK polyubiquitination in the presence of WT ubiquitin. Furthermore, we found TRIM31 substantially increased SYK polyubiquitination in the presence of K27, but not with other ubiquitin mutants (Fig. 1d), indicating TRIM31 mainly promotes K27-linked polyubiquitination of SYK. To validate the ubiquitination of endogenous SYK were induced by TRIM31, we assessed the polyubiquitination of SYK in BMDCs prepared from *Trim31*^*+/+*^ and *Trim31*^*−/−*^ mice upon stimulation with Zymd or α-mannan. The endogenous SYK polyubiquitination was significantly decreased in *Trim31*^*−/−*^ BMDCs as compared with *Trim31*^*+/+*^ BMDCs after stimulation with Zymd or α-mannan (Fig. 1e). Importantly, K27-linked SYK polyubiquitination was decreased, while K48-linked and K63-linked polyubiquitination remained unchanged (Fig. 1e).

SYK has 49 lysine residues, we next investigated which lysine residues of SYK might be targeted by TRIM31 for the K27-linked polyubiquitination. We performed mass spectrometry (MS) analysis and systemically investigated the potential ubiquitinated lysine residues of SYK. A total of seven ubiquitinated lysine residues (K124, K165, K261, K368, K375, K386, K517) were identified in the MS analysis (Supplementary Fig. S3a, b). We then constructed several SYK mutants including K124R, K165R, K261R, K368R, K375R, K386R, and K517R, in which the potential ubiquitinated lysine residues were replaced with arginine. We transfected these SYK mutants into HEK293T cells together with Myc-TRIM31 and found that TRIM31-mediated K27-linked ubiquitination was decreased in K375R and K517R mutants (Fig. 1f). Notably, mutation at both loci (K375R, K517R) substantially attenuated the K27-linked ubiquitination of SYK to the basal level (without TRIM31 catalyzation) (Fig. 1f). Taken together, these results suggest that TRIM31 directly binds with and catalyzes the K27-linked polyubiquitination of SYK at Lys375 and Lys517.

### TRIM31 deficiency impaired anti-fungal immune responses in vivo

Others and we have demonstrated that TRIM31 plays an essential role in the regulation of innate antiviral immunity.^39,47,48^ However, whether TRIM31 plays a critical role in innate antifungal immunity is not known. Given that TRIM31 catalyzes the polyubiquitination of SYK, we proposed that TRIM31 might influence anti-fungal immunity.

**Fig. 2:**
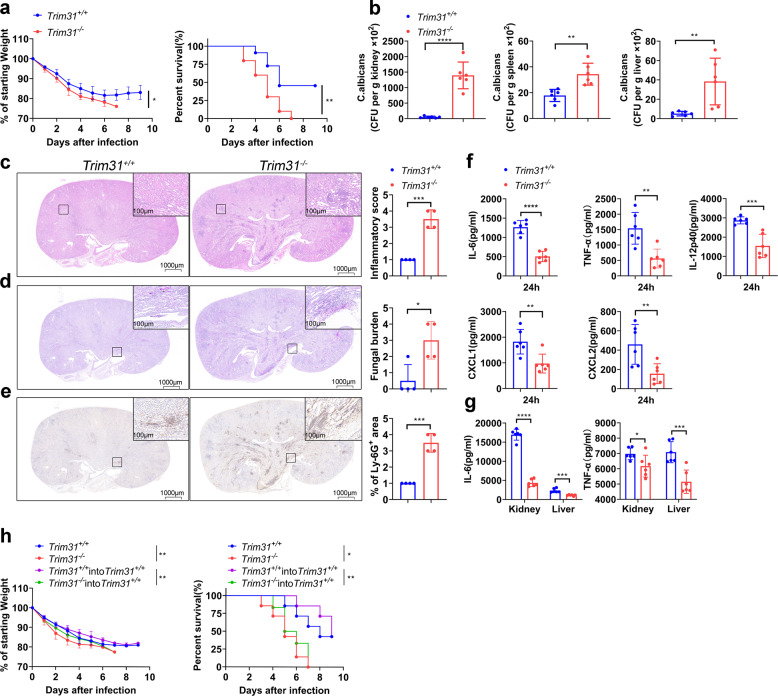
TRIM31 is indispensable for anti-fungal immunity. **a** Weight loss (left) and survival (right) of *Trim31*^+/+^ (*n* = 11), *Trim31*^−/−^ (*n* = 10) male littermates (7–8-week-old) after infection with *C. albicans* strain SC5314 (2 × 10^5^ fungal cells per mouse). **b** Statistics of *C. albicans* in the kidneys, spleens, and livers of *Trim31*^+/+^ and *Trim31*^−/−^ mice infected with *C. albicans* for 5 days, evaluated by serial dilution of homogenized tissues and presented as CFU per gram of the indicated tissue. **c** Representative images of hematoxylin–eosin (HE)-stained kidney sections of *Trim31*^+/+^ and Trim31^−/−^ mice 5 days after systemic *C. albicans* infection, and obtained inflammatory score based on tissue destruction and renal immune cell infiltration. **d** Representative images of periodic acid-Schiff (PAS)-stained kidney sections of *Trim31*^+/+^ and *Trim*31^−/−^ mice 5 days after *C. albicans* infection, and obtained score based on fungal load. **e** Representative images of kidney sections stained for the neutrophil marker Lys-6G^+^ and quantification of kidney area scored as Ly-6G^+^ 5 days after infection of *Trim31*^+/+^ and Trim31^−/−^ mice with *C. albicans*. **f** ELISA analysis of serum cytokines and chemokines from *Trim31*^+/+^ and *Trim31*^−/−^ mice (*n* = 6 per group) 24 h after infection with *C. albicans* (1 × 10^6^ fungal cells per mouse). **g** ELISA analysis of IL-6 and TNF-α from the supernatant of the organ of the *Trim31*^+/+^ and *Trim31*^−/−^ mice 5 days after systemic *C. albicans* infection (2 × 10^5^ fungal cells per mouse). **h** Weight loss (left), as compared to starting weight, and survival (right) over time of lethally irradiated *Trim31*^+/+^ mice that were reconstituted with bone marrow from *Trim31*^+/+^ (*n* = 7) or *Trim31*^−/−^ mice (*n* = 6), recipient mice were intravenous injected with 1 × 10^7^ BM cells. The control group was *Trim31*^+/+^ (*n* = 7) and *Trim31*^−/−^ (*n* = 7). Both of them were subsequently infected with *C. albicans* (2 × 10^5^ fungal cells per mouse) though the tail vein. *P < 0.05; **P < 0.01; ***P < 0.001 and ****P < 0.0001 (two-way analysis of variance in (**a**, **h** left), log-rank test (**a**, **h** right) or Student’s *t*-test (**b–g**)). One representative experiment of three independent experiments is presented (**b–g**, Data are shown as mean ± s.d.). Each point represents a single mouse (**b**, **c–g**). Representative images of *Trim31*^+/+^ (*n* = 4) and *Trim31*^−/−^ (*n* = 4) are shown, three sections per kidney were analyzed, scale bars, 1000 µm, 100 µm (insets) (**c–e**)

To confirm the in vivo function of TRIM31 anti-fungal immune responses, we intravenously infected *Trim31*^*−/−*^ mice and *Trim31*^*+/+*^ mice with a lethal dose of *C. albicans*. The result showed that *Trim31*^*−/−*^ mice were more sensitive to fungal infection than *Trim31*^*+/+*^ mice (Fig. 2a). While *Trim31*^*−/−*^ mice manifested a massive loss of weight after infection with *C. albicans* and finally died of infection, *Trim31*^*+/+*^ mice showed moderate loss of weight, with 45% survival and recovery (Fig. 2a).

To further elucidate the function of TRIM31 in anti-fungal immunity, then we assessed the fungal loads in mice at 5 days after infection. We found *Trim31*^*−/−*^ mice exhibited more *C. albicans* colony forming units (CFU) in the kidney, spleen, and liver, compared to that in *Trim31*^*+/+*^ mice (Fig. 2b). Histopathologic analysis of kidneys demonstrated that *Trim31*^*−/−*^ mice exhibited increased renal inflammation and lots of *C. albicans* yeast cells and hyphae (Fig. 2c, d). Besides, the neutrophils infiltrated into the kidneys of infected *Trim31*^*−/−*^ mice were significantly higher than *Trim31*^*+/+*^ mice (Fig. 2e). Further, we assessed the innate immune response of *C. albicans*-infected *Trim31*^*+/+*^ and *Trim31*^*−/−*^ mice. At early time points of *C. albicans* infection, *Trim31*^*−/−*^ mice showed significantly reduced secretion of IL-6, TNF-α, IL-12, CXCL1, and CXCL2 in their serum at 24 h post-infection as compared with *Trim31*^*+/+*^ mice (Fig. 2f). Consistently, *Trim31*^*−/−*^ mice also had lower levels of IL-6 and TNF-α in the homogenates of kidney and liver after 5 days of infection with *C. albicans* (Fig. 2g).

To investigate the cellular basis of TRIM31 in anti-fungal protective function, we reconstituted lethally irradiated *Trim31*^*+/+*^ mice with syngeneic *Trim31*^*−/−*^ or *Trim31*^*+/+*^ bone marrow (BM) to generate BM-chimeric mice. *Trim31*^*+/+*^ mice reconstituted with *Trim31*^*−/−*^ BM displayed a phenotype similar to that of mice with total *Trim31* deficiency after fungal infection (Fig. 2h), indicating the radiosensitive hematopoietic cells were involved in the anti-fungal immune response. Taken together, these results suggest that TRIM31 positively regulates the anti-fungal immune response in vivo.

### TRIM31 regulates anti-fungal Th1 and Th17 responses

Upon sensing fungal PAMPs, CLRs can induce or modulate Th1 and Th17 responses. We next sought to determine whether TRIM31 regulates Th1 and Th17 responses after *C. albicans* infection.

**Fig. 3:**
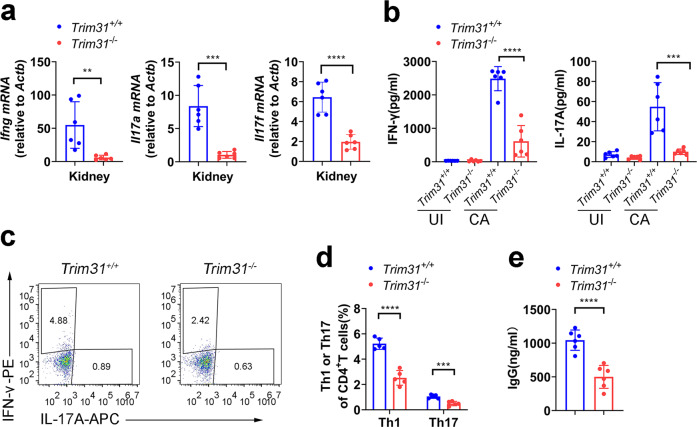
TRIM31 is indispensable for anti-fungal Th1 and Th17 responses. **a** qRT-PCR analysis of *Ifng*, *Il17a*, and *Il17f* from kidneys of *Trim31*^+/+^ (*n* = 6) and *Trim31*^−/−^ (*n* = 6) mice infected with *C. albicans* (2 × 10^5^ fungal cells per mouse) for 5 days. **b** ELISA analysis of IFN-γ (left) and IL-17A (right) in the supernatant of splenic cells obtained from *Trim31*^+/+^ and *Trim31*^−/−^ mice (*n* = 6 per group) infected with *C. albicans* (2 × 10^5^ fungal cells per mouse) for 5 days, followed by stimulation with HKCA-Y (MOI, 1) for 2 days. **c**, **d** Splenic cells isolated from the *Trim31*^+/+^ or *Trim31*^−/−^ mice and stimulated as in (**b**). Intracellular staining of IFN-γ (Th1) and IL-17A (Th17) were determined with flow cytometry. The representative figure is shown in (**c**), and the results are summarized in (**d**). **e** ELISA analysis of IgG in serum from *Trim31*^+/+^ and *Trim31*^−/−^ mice (*n* = 6 per group) infected with *C. albicans* (2 × 10^5^ fungal cells per mouse) for 5 days. *P < 0.05; **P < 0.01; ***P < 0.001; and ****P < 0.0001 (Student’s *t*-test in **a**, **b**, **d**, **e**). Data are from one experiment representative of three independent experiments (**a**, **b**, **d**, **e**; mean ± s.d.)

After 5 days of infection with *C. albicans*, real-time PCR assay showed that expression of the genes encoding *Ifng*, *Il17a,* and *Il17f* was attenuated in the kidneys from *Trim31*^*−/−*^ mice compared to *Trim31*^*+/+*^ mice (Fig. 3a). Further, we isolated spleen cells from *Trim31*^*−/−*^ and *Trim31*^*+/+*^ mice infected with or without *C. albicans*. We found re-stimulation of the spleen cells with HKCA-Y from *Trim31*^*+/+*^ mice infected with *C. albicans* greatly increased the secretion of IL-17A and IFN-γ (Fig. 3b). While the secretion of IL-17A and IFN-γ was greatly attenuated in the spleen cells from infected *Trim31*^*−/−*^ mice upon re-stimulation with HKCA-Y (Fig. 3b). We also investigated the T-cell differentiation in the spleen at day 5 after *C. albicans* infection. The percentage of Th1 and Th17 cells in the spleen was greatly decreased in *Trim31*^*−/−*^ mice compared to that in the spleen from *Trim31*^*+/+*^ mice (Fig. 3c, d).

Antibody responses to fungal infection are identified as playing an important role in immune protection.^49^ *C. albicans* also is the major inducer of anti-fungal immunoglobulin G (IgG),^50^ we found that *Trim31*^*−/−*^ mice showed significantly reduced production of IgG in their serum after 5 days infection as compared with *Trim31*^*+/+*^ mice. Together, these results demonstrate that TRIM31 is required for the Th1 and Th17 differentiation after *C. albicans* infection.

### TRIM31 regulates anti-fungal innate immunity

**Fig. 4:**
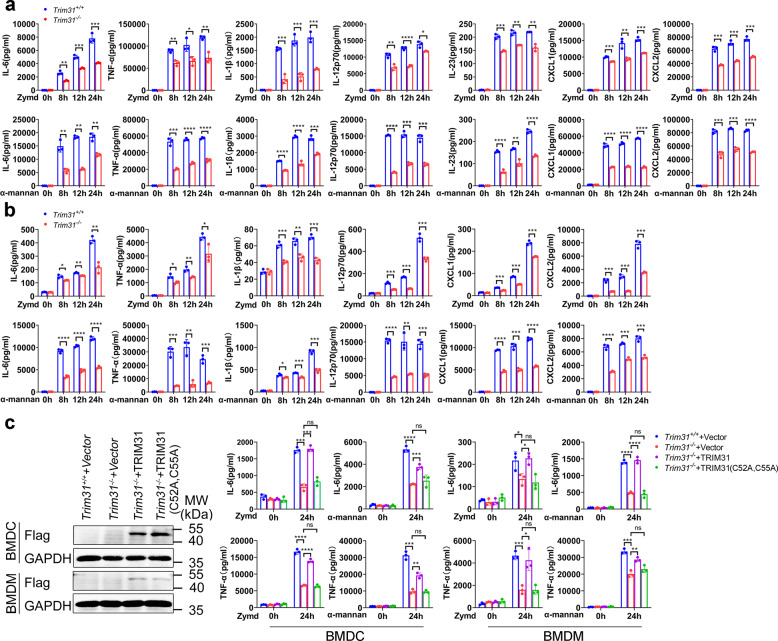
TRIM31 controls antifungal activities of BMDCs and BMDMs. **a** ELISA analysis of IL-6, TNF-α, IL-1β, IL-12, IL-23, CXCL1, and CXCL2 in supernatants of *Trim31*^+/+^ and *Trim31*^−/−^ in BMDCs stimulated with Zymd (100 µg/ml) and α-mannan (100 µg/ml) for the indicated time points. **b** ELISA analysis of IL-6, TNF-α, IL-1β, IL-12, CXCL1, and CXCL2 in supernatants of *Trim31*^+/+^ and *Trim31*^−/−^ in BMDMs stimulated with Zymd and α-mannan for the indicated time points. The ligands and concentrations are the same as (**a**). **c** Immunoblot analysis of the protein expression of TRIM31 in *Trim31*^−/−^ BMDCs and BMDMs transduced as in right (left). ELISA analysis of IL-6 (upper) and TNF-α (lower) in supernatants of *Trim31*^+/+^ and *Trim31*^−/−^ in BMDCs and BMDMs, respectively, transduced by lentiviral vector GV492, GV492-mTRIM31, and GV492-mTRIM31 (C52A, C55A) followed by stimulation with Zymd and α-mannan for 0–24 h (right). *P < 0.05; **P < 0.01; ***P < 0.001; and ****P < 0.0001 (Student’s *t*-test in **a–c**). Experiments were done in triplicates (mean ± s.d. in **a–c**)

SYK is a key adaptor of anti-fungal innate immune response via CLRs. Upon activation, SYK is phosphorylated and modulates the activation of NF-κB and MAPKs, which results in the production of proinflammatory cytokines and chemokines. We next sought to examine the function of TRIM31 in CLR-induced cytokine and chemokine production. Activation CLRs, such as Dectin-1, Dectin-2/3, and Mincle, elicit the production of proinflammatory cytokines and chemokines, which play important roles in anti-fungal innate immunity. We obtained BMDCs from *Trim31*^*+/+*^ and *Trim31*^*−/−*^ mice, followed by stimulation with various CLR ligands Zymd (Dectin-1 ligand), α-mannan (Dectin-2/3 ligand), TDB (Mincle ligand), heat-killed *C. albicans* yeast (HKCA-Y) and heat-killed *C. albicans* hyphae (HKCA-H). We found that the production of proinflammatory cytokines including IL-6, TNF-α, IL-1β, IL-12, IL-23, and chemokines such as CXCL1 and CXCL2 were markedly reduced in *Trim31*^*−/−*^ BMDCs compared to that in *Trim31*^*+/+*^ BMDCs (Fig. 4a and Supplementary Fig. S4a). The deletion of TRIM31 in BMDMs also reduced the production of proinflammatory cytokines including IL-6, TNF-α, IL-1β, IL-12, and chemokines CXCL1 and CXCL2 (Fig. 4b and Supplementary Fig. S4b). Furthermore, we restored TRIM31 protein expression in *Trim31*^*−/−*^ BMDCs and BMDMs through lentiviral infection. WB analysis showed successful restoration of TRIM31 protein expression (Fig. 4c). We found reconstitution of WT TRIM31, rather than the catalytically inactive mutant mTRIM31 (C52A, C55A), was able to restore the induction of IL-6 and TNF-α upon stimulation with Zymd or α-mannan stimulation (Fig. 4c), indicating TRIM31 E3 ligase activity was important for CLR-mediated pro-inflammatory cytokines production. Thus, our results indicate that TRIM31 is a positive regulator for cytokine and chemokine production in response to *C. albicans* infection.

### TRIM31 regulates SYK kinase activity

**Fig. 5:**
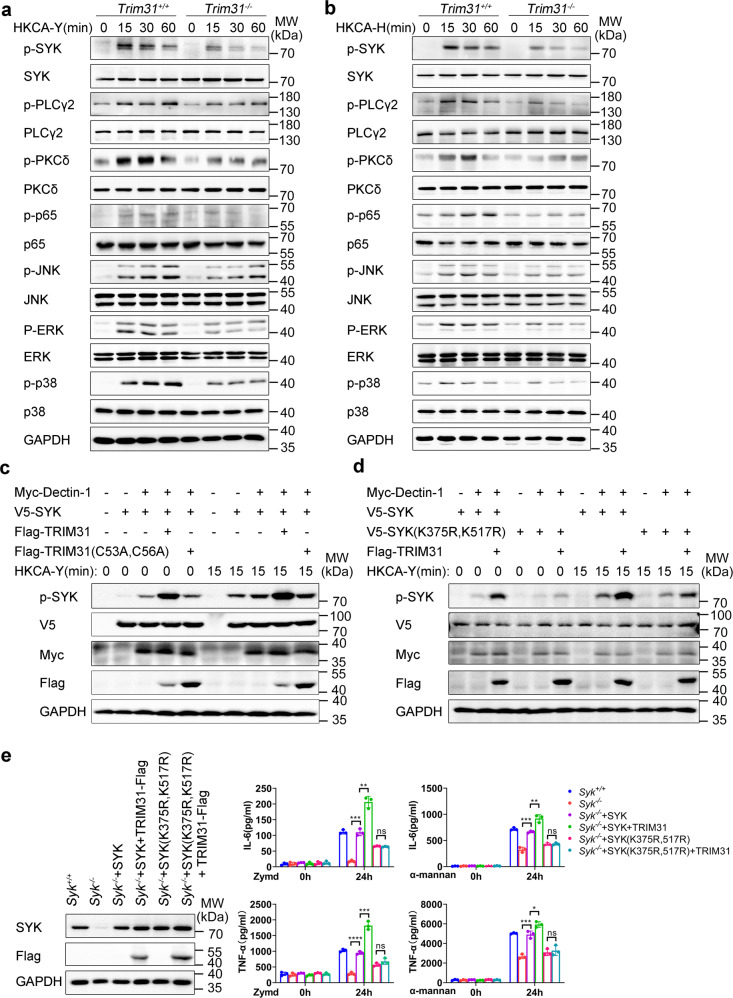
TRIM31 promotes SYK phosphorylation. **a**, **b** Western blot analysis of phosphorylated and total proteins in lysates of *Trim31*^*+/+*^ and *Trim31*^*−/−*^ BMDCs stimulated with HKCA-Y (MOI, 2) (**a**) or HKCA-H (MOI, 1) (**b**) for indicated time points. **c** Immunoblot analysis of SYK phosphorylation and activation in HEK293T cells co-transfected by various combinations of plasmids expressing V5-SYK, Myc-Dectin-1, Flag-TRIM31, and Flag-TRIM31 (C53A, C56A), cells were unstimulated or stimulated by HKCA-Y (MOI, 2) for 15 min. **d** Immunoblot analysis of SYK or SYK (K375R, K517R) phosphorylation and activation in HEK293T cells transfected by various combinations of plasmids expressing V5-SYK, V5-SYK (K375R, K517R), Myc-Dectin-1, and Flag-TRIM31, cells were unstimulated or stimulated by HKCA-Y (MOI, 2) for 15 min. **e** Immunoblot analysis of the protein level of SYK or its mutants in *Syk*^−/−^ BMDCs transduced by the indicated lentiviruses (left). ELISA analysis of IL-6 or TNF-α in *Syk*^−/−^ BMDCs transduced as in the left panel, followed by stimulation for 0–24 h with Zymd or α-mannan (right). ns, not significant (*P* > 0.05); *P < 0.05; **P < 0.01; ***P < 0.001; and ****P < 0.0001 (Student’s *t*-test). Data are representative of three independent experiments (**a–e**)

Based on the results above mentioned, we postulated that TRIM31 may affect the CLR pathways by directly regulating SYK kinase activation. Thus, we prepared BMDCs from *Trim31*^+/+^ and *Trim31*^−/−^ mice, followed by stimulation with HKCA-Y and HKCA-H and measured the phosphorylation (active form) of the CLR-induced signaling. We found that the phosphorylation of SYK in *Trim31*^−/−^ BMDCs was significantly attenuated in response to HKCA-Y and HKCA-H compared to that in *Trim31*^+/+^ BMDCs (Fig. 5a, b). SYK-dependent phosphorylation of PLCγ2 and PKCδ were also attenuated in *Trim31*^−/−^ BMDCs (Fig. 5a, b). Additionally, phosphorylation of the downstream molecular ERK, JNK, and p38 were down-regulated in *Trim31*^−/−^ BMDCs as well. Furthermore, activation of NF-κB signaling indicated by p65 phosphorylation was also decreased in *Trim31*^−/−^ BMDCs (Fig. 5a, b). We obtained similar results in BMDMs (Supplementary Fig. S5a, b). These data indicate that TRIM31 positively regulates SYK-associated signaling cascades in the CLR pathway.

To directly confirm SYK polyubiquitination mediated by TRIM31 is required for SYK activation, Dectin-1, SYK, and WT TRIM31 or TRIM31 (C53A, C56A) were co-expressed in HEK293T cells followed by stimulation with HKCA-Y. Meanwhile, we also transfected Dectin-2, FcRγ, SYK, and WT TRIM31 or TRIM31 (C53A, C56A) into HEK293T followed stimulation with HKCA-H. Overexpression of WT TRIM31, but not TRIM31 (C53A, C56A), greatly enhanced SYK phosphorylation at the basal level, and fungal stimulation further elevated SYK phosphorylation (Fig. 5c and Supplementary Fig. S5c). To determine whether polyubiquitination of SYK at K375 and K517 sites is required for SYK activation, we overexpressed Dectin-1, SYK, SYK (K375R, K517R) mutant, and TRIM31 into HEK293T followed stimulation with fungal stimulation. TRIM31 significantly enhanced the phosphorylation of WT SYK, but not SYK (K375R, K517R) mutant before and after HKCA-Y stimulation (Fig. 5d) or HKCA-H (Supplementary Fig. S5d). These results suggest that the K375/517-linked polyubiquitin chains mediated by TRIM31 is critical for SYK activation.

To investigate whether TRIM31-mediated SYK ubiquitination is involved in antifungal immune responses, we further reconstituted *Syk*^*−/−*^ BMDCs with WT SYK or SYK (K375R, K517R) mutant through lentiviral infection. WB analysis showed the successful reconstitution of SYK protein in *Syk*^*−/−*^ BMDCs (Fig. 5e). As expected, SYK deficiency attenuated Zymd- or α-mannan-induced IL-6 and TNF-α production (Fig. 5e). Reintroduction of SYK or SYK (K375R, K517R) efficiently restored the induction of IL-6 and TNF-α by Zymd or α-mannan in *Syk*^−/−^ BMDCs (Fig. 5e). Notably, TRIM31 greatly increased the production of IL-6 and TNF-α in *Syk*^−/−^ BMDCs reconstituted with WT SYK, whereas SYK (K375R, K517R) mutant-mediated induction of IL-6 and TNF-α was not further increased in the presence of TRIM31 (Fig. 5e). Taken together, these data demonstrate that SYK polyubiquitination at K375 and K517 mediated by TRIM31 is essential for SYK activation and the subsequent pro-inflammatory cytokines production.

### TRIM31-mediated polyubiquitination promotes recruitment of SYK to CLRs

Ligation with CLR agonist and subsequent recruitment of SYK to the receptors leads to the phosphorylation at Y525/526 (Y519/520 in mice) and activation of SYK to initiate the downstream CLR signaling.^51^ To investigate the sequential order of the ubiquitination and phosphorylation of SYK during the process of SYK activation, we constructed an SYK mutant (Y525F, Y526F), in which the phosphorylation tyrosine sites at positions 525 and 526 were mutated to phenylalanine. We found that WT SYK and SYK (Y525F, Y526F) had similar binding capacity with TRIM31 (Supplementary Fig. S6a). Of note, TRIM31 could still mediate the ubiquitination of SYK (Y525F, Y526F) similar to that of WT SYK (Supplementary Fig. S6b). These results suggest that SYK phosphorylation is not required for TRIM31-mediated SYK ubiquitination.

**Fig. 6:**
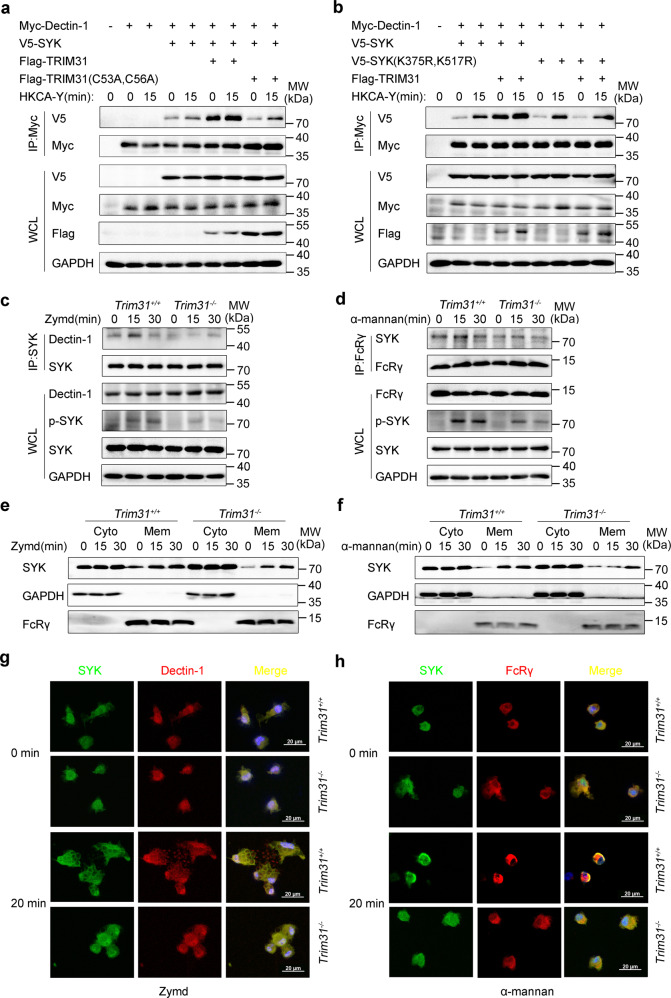
TRIM31 positively regulates translocation of SYK to the membrane and colocalization with Dectin-1 and FcRγ. **a** HEK293T cells were co-transfected with Myc-Dectin-1, V5-SYK, Flag-TRIM31, or Flag-TRIM31 (C53A, C56A) by various combinations, cells were unstimulated or stimulated with HKCA-Y (MOI, 2) for 15 min. Then cell lysates were IP with anti-Myc and WCL, respectively, probed with antibodies (left margins). **b** HEK293T cells were transfected with Myc-Dectin-1, V5-SYK, V5-SYK (K375R, K517R), and Flag-TRIM31 by various combinations, cells were unstimulated or stimulated with HKCA-Y (MOI, 2) for 15 min. Followed by IP with anti-Myc and WCL, respectively, probed with antibodies (left margins). **c**, **d** *Trim31*^+/+^ and *Trim31*^−/−^ BMDCs unstimulated (0 min) or stimulated with Zymd (**c**) or α-mannan (**d**) for 15 or 30 min, followed by IP with anti-SYK or anti-FcRγ, probed with antibodies (left margins). **e**, **f** *Trim31*^+/+^ and *Trim31*^−/−^ BMDCs were stimulated with Zymd (**e**) or α-mannan (**f**) for 15 or 30 min, and whole-cell lysates were separated into membrane and cytosolic fractions. Immunoblot analysis with antibodies (left margins). **g**, **h** Using SYK-conjugated specific antibody (*green*), Dectin-1 or FcRγ conjugated specific antibody (*red*) while the nuclear compartment was observed by DAPI-staining of DNA (*blue*). Scale bars, 20 µm. Data are from one experiment representative of three independent experiments (**a–h**)

Because the binding of SYK to CLRs promotes its phosphorylation/activity, we hypothesized that K27-linked polyubiquitination might affect SYK recruitment to CLRs. To confirm this, HEK293T cells were transfected with Dectin-1, SYK, and TRIM31 or TRIM31(C53A, C56A), then the cells were treated with HKCA-Y or left untreated. Overexpression of WT TRIM31, but not TRIM31 (C53A, C56A), greatly increased the interaction of Dectin-1 and SYK, and fungal stimulation further elevated the association between Dectin-1 and SYK (Fig. 6a). Similarly, the interaction between SYK and FcRγ was also increased in the presence of WT TRIM31, but not TRIM31 (C53A, C56A) in HEK293T cells transfected with Dectin-2, FcRγ, and SYK before or after stimulation with HKCA-H (Supplementary Fig. S6c). These data suggest that the elevated interaction between SYK and Dectin-1/Dectin-2-FcRγ is dependent on TRIM31 catalytic activity.

Because TRIM31-mediated polyubiquitination of SYK at K375 and K517 (Fig. 1f), we hypothesized that K375 and K517 within SYK are important for the recruitment of SYK to Dectin-1. We then transfected Dectin-1, TRIM31, WT SYK, or SYK (K375R, K517R) into HEK293T cells, followed by fungal stimulation or left untreated. We found that TRIM31 greatly increased the interaction between WT SYK and Dectin-1. However, this increased interaction is not observed when SYK was mutated at K375 and K517 (Fig. 6b). Similarly, the interaction between WT SYK and FcRγ was increased in the presence, while the interaction between SYK (K375R, K517R) and FcRγ was unchanged in the presence of TRIM31 (Supplementary Fig. S6d). Further, we showed that Zymd and α-mannan stimulation in BMDCs increased the interaction between SYK and Dectin-1 or FcRγ (Fig. 6c, d). However, Zymd-induced and α-mannan-induced interactions between SYK and Dectin-1/FcRγ were decreased in *Trim31*^*−/−*^ BMDCs compared to that in *Trim31*^*+/+*^ BMDCs (Fig. 6c, d). Thus, our results highlight the important role of TRIM31 in modulating the interaction between SYK and CLR receptors.

### TRIM31 promotes SYK translocation to the membrane

CLRs are a family of transmembrane proteins, but SYK resides predominantly in the cytosol in resting BMDCs. SYK is translocated to the cell membrane, where it interacts with CLRs upon CLR ligation. To independently verify whether TRIM31 promotes SYK recruitment to cell membrane followed stimulation, we prepared cell membrane and cytosolic fractions from BMDCs after stimulation with Zymd or α-mannan. We found these stimulations could induce SYK translocation to the membrane, while SYK membrane translocation was greatly decreased in *Trim31*^*−/−*^ BMDCs (Fig. 6e, f). To directly visualize the translocation of SYK to the cell membrane, confocal microscopy was performed. In resting cells, SYK was found to be diffusely distributed in the cell cytosol of BMDCs, and Zymd or α-mannan stimulation recruited SYK to the cell membrane and colocalized with Dectin-1 or FcRγ. However, the colocalization was decreased in *Trim31*^*−/−*^ BMDCs (Fig. 6g, h). Previous studies have shown that Dectin-1 agonists could induce a rearrangement of the cell membrane and trigger cellular phagocytosis.^52^ We also found Zymd induced fewer phagosomes in *Trim31*^*−/−*^ BMDCs (Fig. 6g). In conclusion, these data suggest that TRIM31-mediated polyubiquitination promotes SYK recruitment to Dectin-1 or Dectin-2-FcRγ.

### TRIM31 inhibits SHP-1-mediated SYK dephosphorylation

**Fig. 7:**
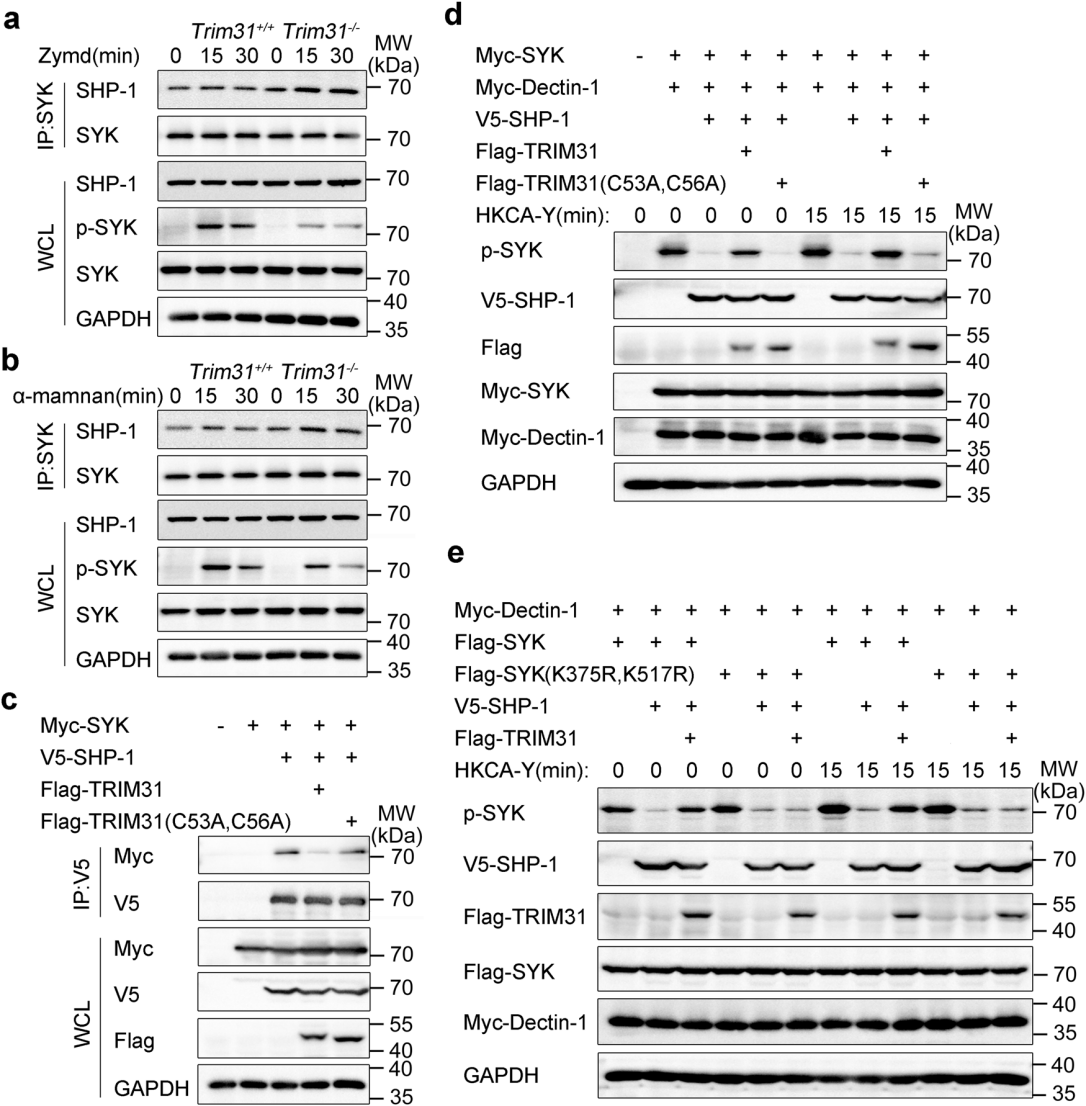
TRIM31 inhibits SHP-1 regulation SYK activation. **a, b** *Trim31*^+/+^ and *Trim31*^−/−^ BMDCs uninfected (0 min) or infected Zymd (**a**) or α-mannan (**b**) for 15 min and 30 min, followed by IP with anti-SYK, probed with anti-SHP-1, WCL immunoblot analysis with indicated antibodies (left margins). **c** HEK293T cells were transfected with Myc-SYK, V5-SHP-1 and Flag-TRIM31, or Flag-TRIM31 (C53A, C56A). Followed by IP with anti-V5, probed with indicated antibodies (left margins). **d** HEK293T cells were transfected with Myc-SYK, Myc-Dectin-1, V5-SHP-1, Flag-TRIM31, or Flag-TRIM31 (C53A, C56A) by various combinations, cells were unstimulated or stimulated with HKCA-Y (MOI, 2) for 15 min, WCL immunoblot analysis with indicated antibodies (left margins). **e** HEK293T cells transfected by various combinations of plasmids expressing Flag-SYK, Flag-SYK (K375R, K517R), Myc-Dectin-1, V5-SHP-1, and Flag-TRIM31, cells were unstimulated or stimulated by HKCA-Y (MOI, 2) for 15 min. WCL immunoblot analysis with indicated antibodies (left margins). Data are from one experiment representative of three independent experiments (**a–e**)

The non-receptor protein tyrosine phosphatases SHP-1 is found to regulate SYK activation negatively by dephosphorylating SYK.^53–55^ To investigate whether TRIM31-mediated SYK ubiquitination affected the binding between SYK and SHP-1, we prepared BMDCs from *Trim31*^*+/+*^ and *Trim31*^*−/−*^ mice and stimulated with Zymd or α-mannan. We found the interaction between SYK and SHP-1 was increased in *Trim31*^*−/−*^ BMDCs compared to that in *Trim31*^*+/+*^ BMDCs (Fig. 7a, b). Meanwhile, we co-expressed SYK, SHP-1, and WT TRIM31 or TRIM31 (C53A, C56A) in HEK293T cells. WT TRIM31, but not TRIM31 (C53A, C56A) attenuated the interaction between SYK and SHP-1 (Fig. 7c). This suggests that the enzymatic activity of TRIM31 is vital for preventing the interaction between SYK and SHP-1.

To further explore whether TRIM31 influences the dephosphorylation of SYK by SHP-1, we co-expressed the abovementioned proteins in HEK293T cells. As expected, transfection of SHP-1 greatly attenuated SYK phosphorylation, while, transfection of WT TRIM31, but not TRIM31 (C53A, C56A), greatly inhibited SHP-1-mediated SYK dephosphorylation (Fig. 7d). This indicated that TRIM31 is essential for sustaining SYK phosphorylation. To study whether polyubiquitination of SYK at K375 and K517 sites is required for SHP-1-mediated dephosphorylation. We also co-expressed Dectin-1, WT SYK, SYK (K375R, K517R), SHP-1, and TRIM31 in HEK293T cells. WT SYK and SYK (K375R, K517R) mutant were dephosphorylated by SHP-1 as expected (Fig. 7e). Overexpression of TRIM31 attenuated SHP-1-mediated WT SYK dephosphorylation, while, TRIM31 did not prevent SHP-1-mediated dephosphorylation of SYK (K375R, K517R) (Fig. 7e). Together, these data highlight that TRIM31-mediated polyubiquitination is essential in sustaining SYK phosphorylation by inhibiting interaction with SHP-1.

**Fig. 8:**
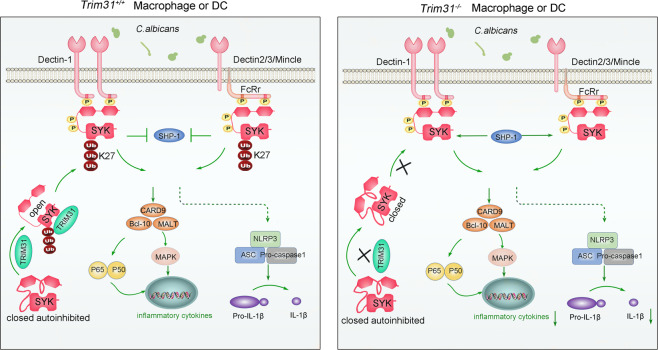
A hypothetical model of the role of TRIM31 in anti-fungal immunity response. TRIM31 catalyzes the K27-linked polyubiquitination of SYK, which can disrupt the autoinhibited status of SYK, promote SYK binding to CLRs, decrease the association of SYK and SHP-1, leading to the increased SYK kinase activity (left). TRIM31 deficiency leads less SYK translocation to the membrane, produces lower amounts of pro-inflammatory cytokines and chemokine in response to *C. albicans* infection (right)

## Discussion

As a key regulator in mounting immune responses against fungal infection, SYK activation is tightly regulated by phosphorylation of the dual tyrosine residues in the linker and kinase domains.^16^ In resting state, the two N-terminal SH2 domains of SYK are distorted in a way preventing its binding with the ITAM motif of Dectin-1/FcRγ, thus SYK is kept in an inactive state. Upon the engagement with β-glucan-containing particles, the cognate receptor Dectin-1 becomes phosphorylated immediately at its ITAM motif, which is believed to be initiated through the Src tyrosine kinase. Then SYK is recruited to the ITAM motif, and further phosphorylates other SYK molecules. The downstream CLR-signaling further activates the downstream CLR-signaling cascades.^56^ However, whether SYK activity is modulated by other post-translational modifications, such as ubiquitination, has not been studied extensively yet.

Here, we identified that TRIM31 functions as an essential regulator for SYK action and the resultant innate antifungal immunity. Mechanistically, TRIM31 specifically catalyzed SYK at K375 and K517 for K27-linked polyubiquitination, which promoted SYK binding to the ITAM motifs of Dectin-1 and FcRγ, and decreased the association with SHP-1, thus facilitated SYK phosphorylation. In addition, *Trim31*-deficient BMDCs and BMDMs showed significantly reduced production of pro-inflammatory cytokines and decreased CLR-mediated signaling activation (Fig. 8). Consistently, *Trim31*-deficient mice were more sensitive to fungal infection than *Trim31*^*+/+*^ mice. To our knowledge, this is the first report defining the critical role of SYK polyubiquitination in the regulation of SYK activation and antifungal immunity.

Protein ubiquitination is a way involved in regulating the proteolysis of SYK. The E3 ligases CBL-B associates with SYK and ubiquitinates SYK for K48-linked polyubiquitination, the ubiquitinated SYK is subsequently recruited to lysosomes for degradation. However, only the phosphorylated SYK can be ubiquitinated and degraded by CBLB as reported.^26^ Instead, our study identified that the E3 ligase TRIM31 interacts and catalyzes K27-linked polyubiquitination of SYK at K375 and K517 upon fungal stimulation. Interestingly, SYK ubiquitination occurs ahead of phosphorylation. And the K27-linked SYK polyubiquitination promotes its translocation to the plasma membrane, which may further amplify the SYK phosphorylation. Thus, our results demonstrate that TRIM31 functions are completely different from CBLB by catalyzing K27-polyubiquitination, and this modification positively regulates SYK -associated downstream signaling activation.

We identified K375 and K517 within SYK as the ubiquitination sites mediated by TRIM31. Ubiquitination at these two lysine residues is important for SYK interaction with CLRs, membrane translocation, and inhibition of binding with SHP-1. K375 localizes close to Tyr348 and Tyr352 within the interdomain B, whose phosphorylation is essential for the disruption of the inactive conformation of SYK and results in kinase activation. K517 is close to Tyr525 and Tyr526, which localize in the activation loop of SYK. Since K27-linked polyubiquitination of SYK at these two sites (K375, K517) promotes its phosphorylation/kinase activity, we propose that polyubiquitination might facilitate the shift of SYK conformation from an inactive status to the active status with the kinase domain exposed.

Previous researches have shown that the members of the TRIM family are greatly involved in a broad range of biological processes, such as the life cycle of HIV, tumor development, and progression.^57^ However, the role of the TRIM family in anti-fungal immunity is less reported. TRIM62 is the only member found to be involved in antifungal immunity through catalyzing CARD9 polyubiquitination at K125 and impacting its activation and the subsequent fungal clearance.^45^ Notably, our study extends the current appreciation of this family in antifungal responses by elucidating the significance of TRIM31 in modulating SYK activation. We performed a screening of this family on the role of ubiquitinating SYK and found that TRIM31 specifically interacts and ubiquitinates SYK. Interestingly, several members such as TRIM26, TRIM40, TRIM65, which have previously been reported to be responsible for anti-viral responses by regulating Interferon-β production,^32,37,46^ are not involved in SYK post-modification. In innate antiviral immune, TRIM31 has been reported as an enhancer to promote aggregation and activation of MAVS.^34^ Based on our findings, TRIM31 is also as a positive factor to regulate innate antifungal immune. So, we think the protective effect of TRIM31 in antiviral and antifungal is consistent. TRIM31 can bind with different adaptor proteins to induce more pro-inflammatory cytokines or interferon through different pathways against fungal pathogens or virus infection in innate immune cells. Collectively, our results indicate that members of the TRIM family play distinct and exclusive roles in combating various pathogens, and TRIM31 is essential for fighting against fungal pathogens via catalyzing K27-linked SYK polyubiquitination.

The previous report has shown that TRIM31 promotes Atg5/Atg7-independent autophagy in a palmitoylation-dependent manner, and eliminates the invading bacteria in intestinal cells.^58^ Similarly, autophagy is a critical tool for the killing of fungal pathogens. Fungal pathogens are recognized by CLRs and are engulfed, thus trigger activation of SYK, ultimately result in the recruitment of the microtubule-associated protein 1A/1B-light chain 3 (LC3)-conjugation machinery consisting of the Atg5 and Atg12. Moreover, fungal pathogens also trigger the LC3-associated phagocytosis pathway.^59^ As noted previously, TRIM31 may promote the elimination of invading fungal through autophagy, besides promoting K27-linked polyubiquitination of SYK. Of course, we need to confirm this function in the future.

In summary, we demonstrated the crucial role of TRIM31 in anti-fungal responses by catalyzing K27-linked polyubiquitination of SYK at K375 and K517. This facilitates the recruitment of SYK to the phosphorylated ITAMs of upstream CLR receptors for SYK activation. It also decreases the association of SYK and SHP-1 to sustain SYK activity. Overall, our work elucidates the mechanisms by which TRIM31 modulates SYK activity, and also highlights the importance of SYK K27-linked ubiquitination in antifungal immunity.

## Materials and methods

### Mice

The *Trim31*^−/−^ mice were described previously.^34^ For the genotyping of the *Trim31*^−/−^ mice, genomic DNA was isolated from tail tips and was validated by sequencing of the RT-PCR fragments (250 bp) in the TALEN-targeting region with the primers (Supplementary Table S3). The homozygous *SYK*^fl/fl^ were hybridized with homozygous tamoxifen-inducible Cre recombinase-estrogen receptor T2 mice (The Jackson Laboratory) to generate *Cre*^*ERT*^*SYK*^*fl/fl*^ for experimentation (obtained from Dr. Hui Xiao, Institute Pasteur of Shanghai, CAS, Shanghai, China). *Cre*^*ERT*^*SYK*^*fl/fl*^ mice were treated with tamoxifen (0.1 ml, 10 mg/ml in corn oil) by intraperitoneal injection every day for 5 consecutive days to induce SYK deletion. There is a 7-day waiting period between the final injection and histological analysis. All the mice were bred and maintained in a pathogen-free animal facility with the approval of the Scientific Investigation Board of the Medical School of Shandong University. All the mice experiments were carried out following the general guidelines published by the Association for Assessment and Accreditation of Laboratory Animal Care.

### Reagents and antibodies

Zymosan delete (Zymd) and TDB were purchased from Invivogen. α-mannan and tamoxifen were purchased from Sigma. Corn oil was from MedChemExpress. IL-4 and GM-CSF were from R&D Systems. Neomin sulfate and Polymyxin B Sulfate were purchased from Solarbio. For transfection into HEK293T, Lipofectamine 3000 reagents (Invitrogen) or Lipofectamine 2000 reagents (Invitrogen) were used. The detailed reagents information was listed in Supplementary Table S4. For immunoblot analysis, the horseradish-peroxidase-conjugated secondary antibodies were from Beijing Zhongshan Jinqiao Biological Technology; antibodies to GAPDH was from Abways technology; protein A/G–agarose was from Santa Cruz Biotechnology; IgG, GFP, p-SYK (Tyr525/Tyr526), SYK, p-PKCδ (Thr505), PKCδ, p-PLCγ2(Tyr759), PLCγ2, p-p65(Ser536), p65, p-JNK1/2 (Thr183/Tyr185), JNK1/2, p-ERK1/2(Thr202/Tyr204), ERK1/2, and p-p38(Thr180/Tyr182), p38 were from Cell Signaling Technology; antibodies to TRIM31, Flag were from Sigma-Aldrich; antibodies to Myc and HA were obtained from Origene; antibody to V5 was from Absin. For immunofluorescent staining, Alexa Fluor 568-conjugated goat anti-rabbit IgG (H + L) and Alexa Fluor 488–conjugated goat anti-Mouse IgG (H + L) were obtained from Invitrogen. For flow cytometry, antibodies to mouse CD3 (17A2), CD4 (GK1.5), IFN-γ (XMG1.2), IL-17A (TC11-18H10.1), and Cell Activation Cocktail (with Brefeldin A) were from BioLegend. All of the detailed information on the antibodies was listed in Supplementary Table S1.

### Plasmids and molecular cloning

The recombinant vectors expressing Myc-tagged human MAVS, Flag-tagged or GFP-tagged human TRIM31 and its mutants had been described.^34^ The recombinant vectors encoding SYK and its mutants were constructed by PCR-based amplification of cDNA from THP1 cells by various sets of primers and then cloned into the pcDNA3.1(+) or pLVX-mCherry. PKCδ plasmid was generated by PCR-based amplification of cDNA from THP-1 cells and subsequently cloned into the pCMV-N-Myc eukaryotic expression vector (Beyotime, China). TRIM10, TRIM40, TRIM62, CARD9, Dectin-2, Dectin-3, Mincle, and BCL10 expression plasmid was constructed by PCR-based amplification of cDNA from THP-1 cells, subsequently cloned into the pcDNA3.1(+). To construct the lentivector-expressing SYK, SYK WT, or mutants were subcloned into the pLVX-IRES-Puro lentivector. All of the primers used for PCR are listed in Supplementary Table S2. Dectin-1, PLCγ2, and FcRγ expression plasmid were bought from GeneCopoeia (Rockville, MD, USA). The vectors for HA-Ub (WT, K48, K63) were from Dr. Hui Xiao (Institute Pasteur of Shanghai, CAS, Shanghai, China). Expression vectors for TRIM14, TRIM27, TRIM38, TRIM39, and TRIM65 were from Dr. Jun Cui (Sun Yat-sen University, Guangzhou, China). Point mutants of SYK were generated using the KOD-Plus-Mutagenesis kit (Toyobo, Osaka, Japan). Primers for mutagenesis PCR are listed in Supplementary Table S2. All plasmids were confirmed by DNA sequencing (The Beijing Genomics Institute).

### Culture and heat inactivation of *C. albicans* strain SC5314

*C. albicans* strain SC5314 was provided by Dr. Changbin Chen (Institute Pasteur of Shanghai, CAS, Shanghai, China). Single colonies of *C. albicans* strain SC5314 from yeast-peptone-dextrose (YPD: Y1375, Sigma) agar plates were inoculated into YPD medium by culture overnight at 30 °C. Yeast cells were cultured at 37 °C for 3 h in YPD medium plus 10% FBS to obtain hyphae. Yeast or Hyphae cells were washed three times and resuspended in PBS buffer, then were incubated at 65 °C for 1 h to kill cells, then obtained HKCA-Y or HKCA-H. The death of Heat-killed yeast or Hyphae cells was verified by plating cells on YPD agar plates.

### *C.* albicans infection

*C. albicans* strain SC5314 was grown on a YPD agar plate overnight at 30 °C. For every infection, a single colony was isolated from an agar plate and grown in YPD media for 24 h at 30 °C. Then yeast cells were washed three times in PBS buffer and adjusted to 2 × 10^5^ or 1 × 10^6^ yeast cells in 0.1 ml of PBS buffer. Yeast cells were injected intravenously into 7- to 8-week-old littermates. Recorded weight loss and survival every day. After 5 days, the kidney, liver, and spleen were collected and measured fungal burden. Homogenized tissues were appropriately diluted and plated on YPD agar. After 24 h, fungal colony-forming units (CFU) were counted.

### Histopathology

For histological analysis, according to standard procedures, the kidneys were fixed in 4% paraformaldehyde solution, embedded in paraffin, and sectioned. 3 µm-thick sections were stained with hematoxylin and eosin (H&E), periodic-acid-Schiff (PAS), or anti-Ly-6G (Servicebio; GB11229). The score of renal inflammation and the intralesional fungal burden has been described previously.^60^ The extent of infiltration by Ly-6G^+^ cells in affected kidneys was evaluated by Caseviewer software.

### Bone marrow transplantation

Acidified water containing polymyxin B sulfate (60,000 U/l) and neomycin (100 mg/l) in filter-top cages to feed age-matched recipient *Trim31*^+/+^ mice, 2 weeks before and 3 weeks after bone marrow transplantation. Then recipient mice were subjected to lethal irradiation with 8 Gy to destroy bone marrow stem cells. Then obtained fresh donor bone marrow cells from *Trim31*^+/+^ and *Trim31*^−/−^ littermates. For transplantation, recipient mice were intravenously injected with 1 × 10^7^ bone marrow cells. After 4 weeks, *C. albicans* strain SC5314 was injected intravenously into recipient mice.

### Generation of BMDMs and BMDCs

Bone marrow cells were harvested from the tibias and femurs of 7–8-week-old mice with RPMI-1640 medium. Then red blood cells were lysed with lysis buffer (solarbio). For the generation of BMDMs, the cells were grown in culture dishes in the presence of M-CSF (secreted by L929 cells), bone marrow cells were cultured in RPMI-1640 medium contained 10% FBS and 15% medium conditioned with L929. On day 5, removed nonadherent cells and added fresh medium containing 10% FBS and 15% medium conditioned with L929. After 7 days of culturing, flow cytometry analysis indicated that 90% CD11b^+^ F4/80^+^cells were harvested. For the generation of BMDCs, bone marrow cells were cultured in RPMI-1640 medium containing 10% FBS, GM-CSF (20 ng/ml), and IL-4 (10 ng/ml). On day 2, removed nonadherent cells and added fresh medium containing 10% FBS, GM-CSF, and IL-4, then changed half fresh medium containing 10% FBS, GM-CSF, and IL-4 every 2 days, at day 9, collected nonadherent cells and resuspended in fresh medium containing GM-CSF for use, flow cytometry analysis indicated that 90% CD11c^+^ were harvested. All the medium contained penicillin (100 U/ml), streptomycin (100 µg/ml).

### RNA quantification

Based on the manufacturer’s instructions (Aidlab Biotech), total RNA was extracted from kidneys or cells, cDNA was reverse-transcribed from total RNA with a PrimeScript RT-PCR Kit (Takara). Supplementary Table S2 included all primers used for RT-PCR assays. Reverse-transcribed samples were amplified by CFX Connect Real-Time PCR Detection System (BIO-RAD) using the SYBR Green PCR Master Mix (Roche), *actin* was used as an internal control, used the 2^−∆∆Ct^ method to calculate relative expression changes.

### ELISA

Based on the manufacturer’s instructions, the concentrations of mouse IL-6, TNF-α, IL-12P40, IL-12P70, IL-1β, IL-17A, and IFN-γ in tissue homogenates or cell culture supernatants were determined by ELISA Kits (Dakewe Biotech). The concentrations of mouse IL-23, CXCL1, CXCL2, and IgG in cell culture supernatants or serum were determined by ELISA Kits (Multi sciences).

### Immunofluorescence staining and confocal analysis

For immunofluorescence staining, cells were fixed with paraformaldehyde (4%, Beyotime) for 20 min, then permeabilized using Triton X-100 (0.1%, Beyotime) for 20 min and blocked for 1 h in 1% BSA (Beyotime) at room temperature. Then the cells were incubated with indicated primary antibodies (Supplementary Table S1) overnight at 4 °C, then cells were washed in wash buffer (Beyotime) four times and incubation with Alexa Fluor 568 (Invitrogen) or Alexa Fluor 488 (Invitrogen) secondary antibodies for 1 h at room temperature, after washing in wash buffer for four times, cells were counterstained with DAPI (Abcam). Cells were imaged with confocal laser microscopy (LSM780, Carl Zeiss).

### Immunoblot, immunoprecipitation, and ubiquitination assay

For immunoblot analysis, cells were lysed with Cell Lysis Reagent (sigma) supplemented with a phosphatase inhibitor (Roche) and protease inhibitor cocktail (sigma). A bicinchoninic acid assay (Thermo fisher) was used to measure the protein concentrations in the extracts, then the protein concentrations were made equal in different samples with cell lysis. For immunoprecipitation (IP), whole-cell extracts were collected in IP buffer, which contained 1% (vol/vol) NP-40, 50 mM EDTA, 150 mM NaCl, 50 mM Tris–HCl (pH 7.4) and a protease inhibitor cocktail (Sigma). After centrifugation for 15 min at 14,000×*g*, supernatants were collected and were made equal in different samples, incubated with 1–2 µg of the corresponding antibodies for 6 h, then Protein A/G beads (Santa Cruz Biotechnology) were added to the supernatants at 4 °C and incubation for 2 h, beads were washed five times with IP buffer at 4 °C. Proteins were eluted with a 2× protein loading buffer from the beads. Then being boiled for 10 min, all the samples were fractionated by 10% SDS–PAGE and were transferred to a PVDF membrane and then probed with the specific antibodies. For analysis of the ubiquitination of SYK or other molecules in HEK293T cells, HEK293T was transfected with HA-Ub (WT) or HA-Ub mutants, Myc-SYK and Flag-TRIM31, and then the cell lysates were immunoprecipitated with anti-Myc and analyzed by immunoblot with anti-HA antibody. For analysis of the ubiquitination of SYK in BMDCs, BMDCs were stimulated with α-mannan or Zymd, then cell lysates were immunoprecipitated with anti-SYK and analyzed by immunoblot with anti-ubiquitin (WT, K27, K48, K63).

### In vitro binding assay

SYK and TRIM31 proteins were purchased from (GeneCopoeia), then mixing SYK and TRIM31 together, followed by IP with anti-TRIM31 and WB with anti-SYK.

### Cell fractionation

BMDCs were stimulated with α-mannan or Zymd, then cells were performed by membrane protein extraction kit (Thermo Scientific), following standard protocols recommended by the manufacturer.

### Lentivirus preparation and infection

The lentiviral vector GV492 expressing GV492-mTRIM31 or GV492-mTRIM31(C52A, C55A) was transiently transfected into HEK293T by mixed packaging plasmids pHelper1.0 and pHelper2.0 (Genechem), and virus-containing cell medium was harvested in 48 h. The pLVX-Myc-SYK recombinant plasmid or mutants was also transfected into 293T cells by mixed packaging plasmids psPAX2 and pMD2G to obtain recombinant lentivirus carrying Myc-SYK or mutants. BMDMs or BMDCs were transduced with Lentivirus supernatant by spin inoculation (650×*g* for 1 h). Cells were sorted based on the expression of GFP by confocal laser microscopy, then gene-expression analysis and cytokine production were performed.

### Splenic T cell responses

Mice were injected intravenously with 2 × 10^5^ *C. albicans* train SC5314 yeast. After 5 days, splenocytes were obtained and stimulated with heat-killed *C. albicans* (MOI, 1) for 48 h. For cytokine detection, supernatants were collected to measure the concentrations of IFN-γ and IL-17A by ELISA. For intracellular staining, cells were stimulated with Cell Activation Cocktail (with Brefeldin A) (Biolegend) for the final 6 h of incubation and then were treated with a Cytofix/Cytoperm kit (eBioscience). The expression of intracellular factors was analyzed by flow cytometry.

### Detection of protein ubiquitination by mass spectrometry and data analysis

For detection of ubiquitination sites of targeted proteins, HEK293T was transfected with HA-Ub (WT), Myc-SYK, and Flag-TRIM31, then the whole-cell lysates were immunoprecipitated with anti-Myc. Protein A/G agarose beads were added to incubation for 2 h. Beads were washed three times with IP buffer at 4 °C, then boiled and loaded to SDS–PAGE. Then Coomassie Blue staining, the specific bands were cut and then analyzed in tandem mass spectrometry (PTM BIOLABS). The resulting MS/MS data were processed and searched against SYK database by Proteome Discoverer 1.3.

### Statistical analysis

Data are given as the mean ± s.d. Comparison of means to identify differences between groups was performed using the Student’s unpaired *t*-test or two-way ANOVA test. The log-rank (Mantel–Cox) test was applied for the survival curves and analyzed with GraphPad Prism 8.0. *P* < 0.05 was considered significant. No data points or mice data were excluded from the study. The study did not use randomization or blinding methods.

## Supplementary information

TRIM31 facilitates K27-linked polyubiquitination of SYK to regulate antifungal immunity

## Data Availability

All data that support the findings of this study are available from the corresponding author upon reasonable request.
