# Supplementary material for: TRIM31 facilitates K27-linked polyubiquitination of SYK to regulate antifungal immunity
Source: Signal Transduct Target Ther. 2021 Aug 6;6:298. doi: 10.1038/s41392-021-00711-3 (PMC8342987; doi:10.1038/s41392-021-00711-3)
Supplement: Supplementary file 1 — TRIM31 facilitates K27-linked polyubiquitination of SYK to regulate antifungal immunity [file 41392_2021_711_MOESM1_ESM.docx]

Supplementary Materials for

TRIM31 facilitates K27-linked polyubiquitination of SYK to regulate antifungal immunity

Xueer Wang^1#^, Honghai Zhang^1#^, Zhugui Shao^1^, Wanxin Zhuang^1^, Chao Sui^1^, Feng Liu^1^, Xiaorong Chen^1^, Jinxiu Hou^1^, Lingli Kong^1^, Hansen Liu^1^, Yi Zheng^1^, Bingyu Liu^1^, Tian Chen^2^, Lei Zhang^1^, Xinming Jia^3^, Chengjiang Gao^1,*^

Correspondence to: cgao@sdu.edu.cn

**This PDF file includes:**

Figures. S1 to S6

Tables S1 to S4


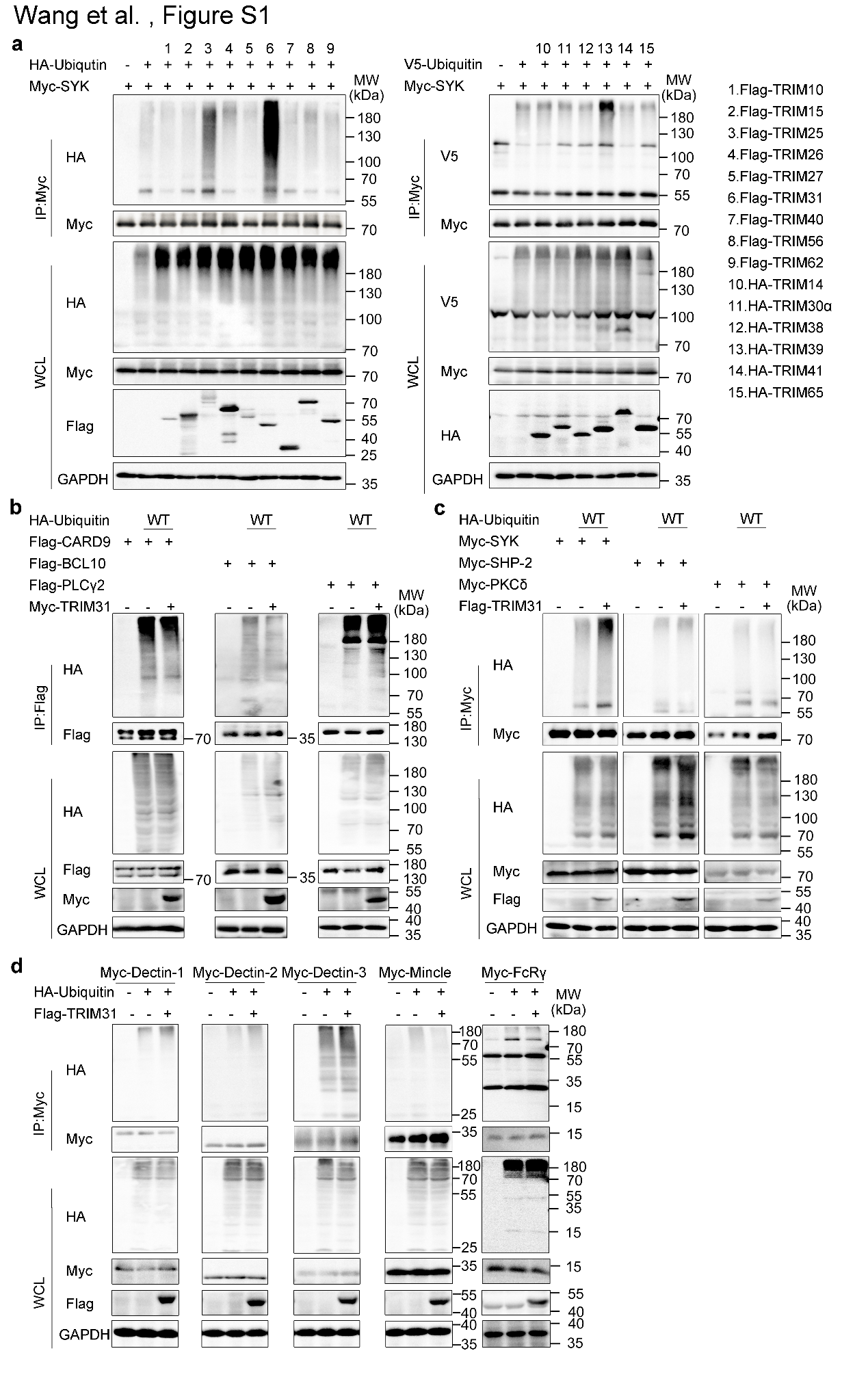


**Figure. S1. TRIM31 regulates polyubiquitination of SYK.** (**a**) Co-immunoprecipitation analysis of the ubiquitination of SYK in HEK293T cells transfected with HA-ubiquitin (WT), Flag-TRIM10, Flag-TRIM15, Flag-TRIM25, Flag-TRIM26, Flag-TRIM27, Flag-TRIM31, Flag-TRIM40, Flag-TRIM56 and Flag-TRIM62 (left). Co-IP analysis of the ubiquitination of SYK in HEK293T cells transfected with V5-ubiquitin (WT), HA-TRIM14, HA-TRIM30α, HA-TRIM38, HA-TRIM39, HA-TRIM41 and HA-TRIM65 (right). (**b,c**) Co-immunoprecipitation analysis of the ubiquitination of CARD9, BCL10, PLCγ2, SYK, SHP-2 and PKCδ in HEK293T cells transfected various combinations with HA-ubiquitin (WT) and Flag-TRIM31 or Myc-TRIM31. (**d**) Co-immunoprecipitation analysis of the ubiquitination of the receptors (Dectin-1, Dectin-2, Dectin-3, Mincle) and the adaptor FcRγ in HEK293T cells transfected with HA-ubiquitin (WT) and Flag-TRIM31. Antibodies (left margins). Data are from one experiment representative of three independent experiments (**a-d**).


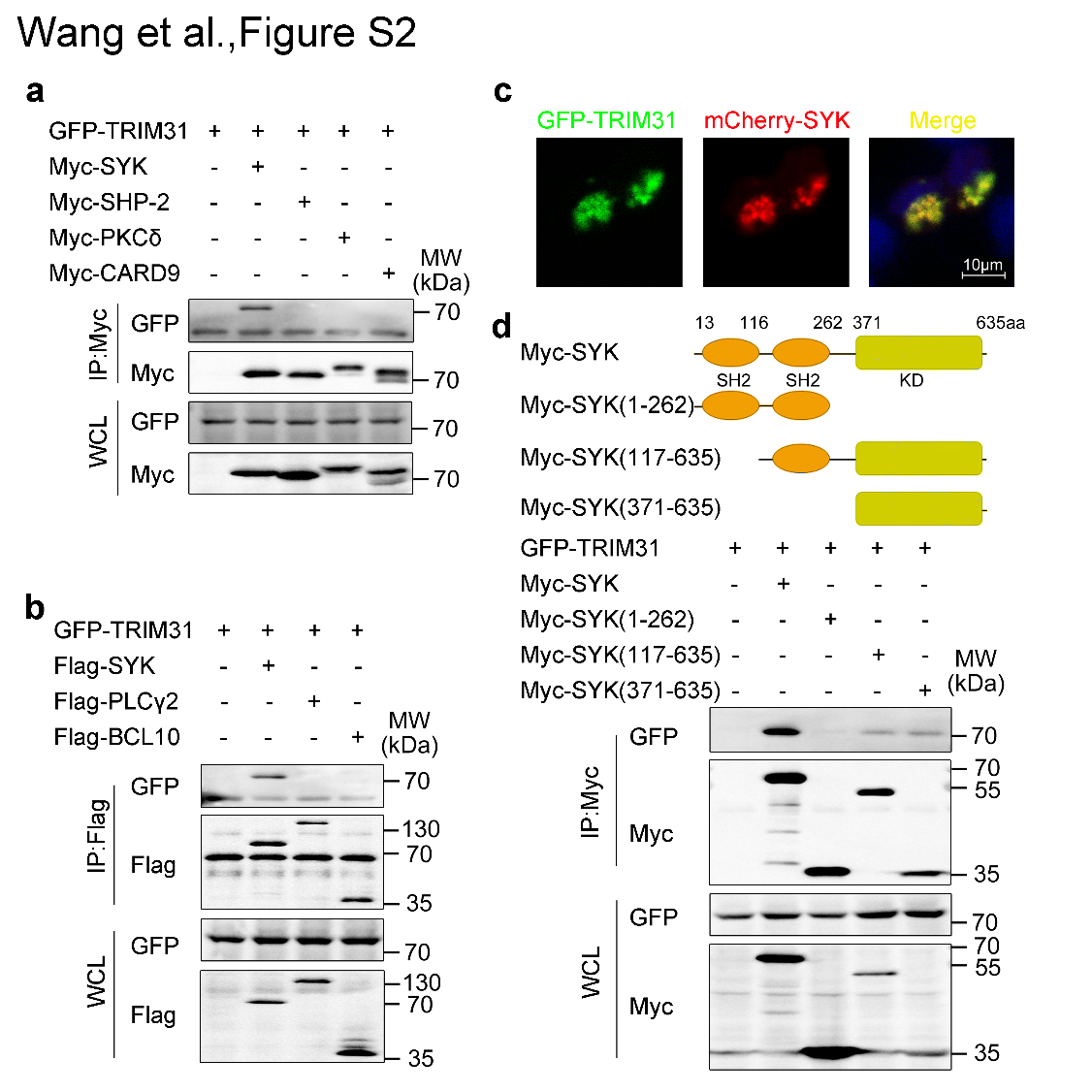
**Figure. S2. TRIM31 targets SYK.** (**a,b**) Co-IP analysis of the interaction between TRIM31 and signaling molecules (SYK, SHP-2, PKCδ, CARD9, PLCγ2 and BCL10) in CLR pathways in HEK293T cells transfected with the indicated expression plasmids. (**c**) Confocal microscopy analysis of relative localization of TRIM31 and SYK in HEK293T transfected with plasmids expressing GFP-TRIM31 and mCherry-SYK for 24h. Scale bars, 10 µm. (**d**) Schematic representation of SYK or SYK truncations (top). Co-IP analysis of the interaction between GFP-TRIM31 and Myc-SYK or SYK truncations in HEK293T cells. Antibodies (left margins). Data are representative of three independent experiments (**a-d**).


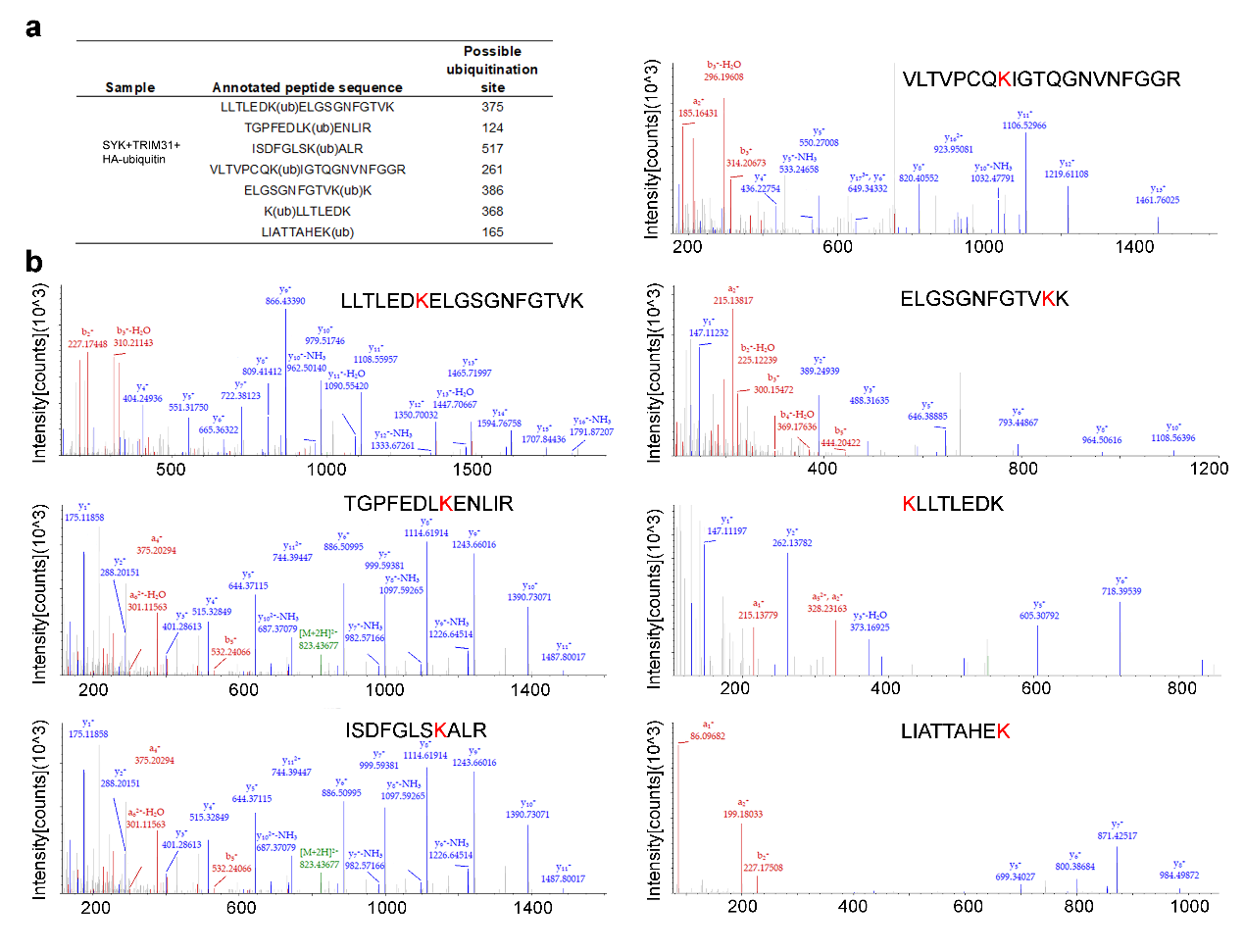


**Figure. S3. TRIM31 promotes K27-linked polyubiquitination of Lys375 and Lys517 on SYK.** (**a,b**) MS analysis of SYK-specific band cut from SDS-PAGE gel.


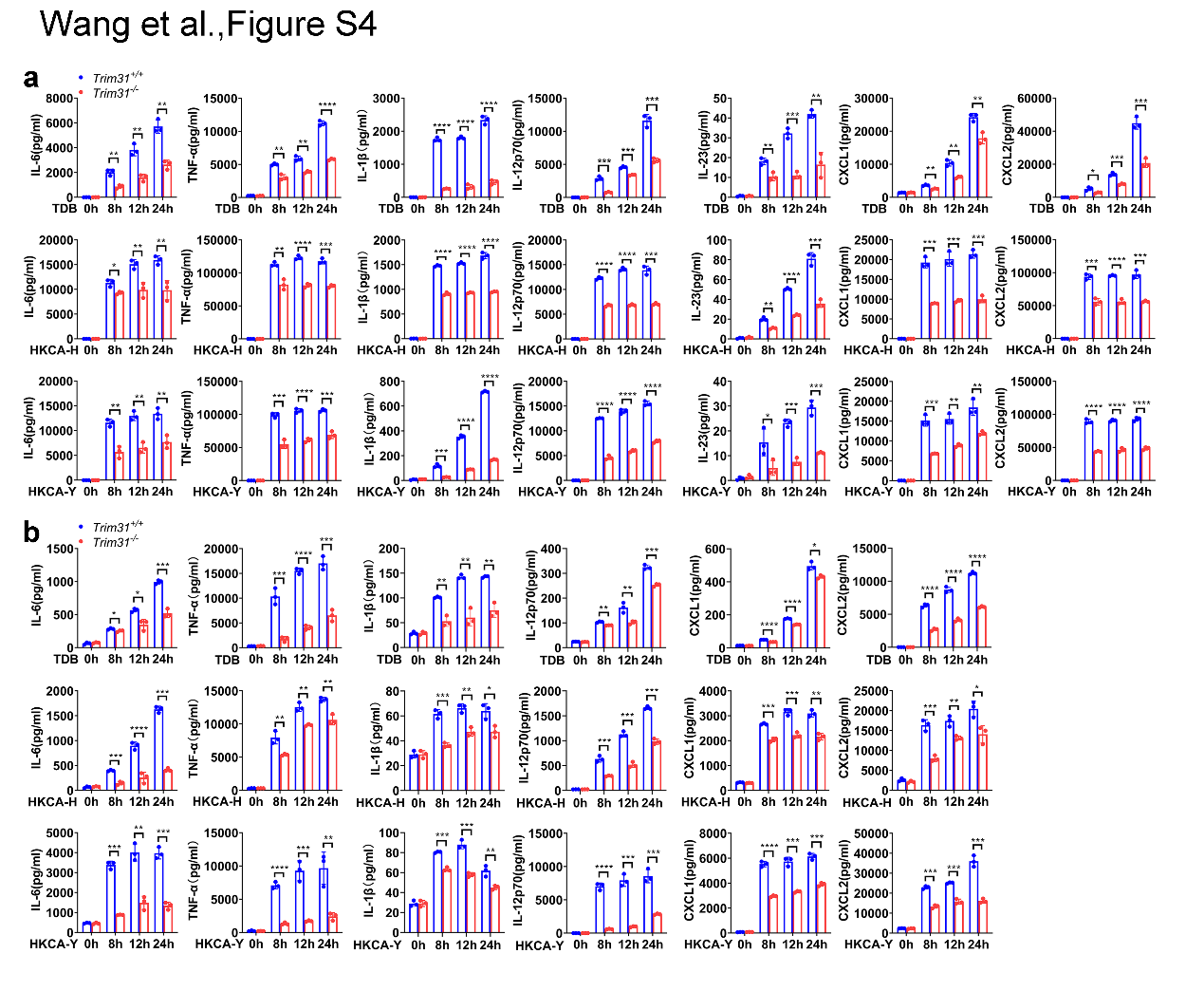


**Figure. S4. TRIM31 regulates Pro-inflammatory cytokines and chemokines production in BMDCs and BMDMs.** (**a**) ELISA analysis of IL-6, TNF-α, IL-1β, IL-12, IL-23, CXCL1 and CXCL2 in supernatants of *Trim31^+/+^* and *Trim31^−/−^* in BMDCs stimulated with TDB (50 µg/well), HKCA-H (MOI, 1) and HKCA-Y (MOI, 2) for indicated time points. (**b**) ELISA analysis of IL-6, TNF-α, IL-1β, IL-12, CXCL1 and CXCL2 in supernatants of *Trim31^+/+^* and *Trim31^−/−^* in BMDMs stimulated with TDB (50 µg/well), HKCA-H (MOI, 1) and HKCA-Y (MOI, 2) for indicated time points. **P* < 0.05, ***P* < 0.01, ****P* < 0.001 and *****P* < 0.0001 (Student’s t-test in **a,b**). Data are from one experiment representative of three independent experiments (**a,b**, mean ± s. d.).


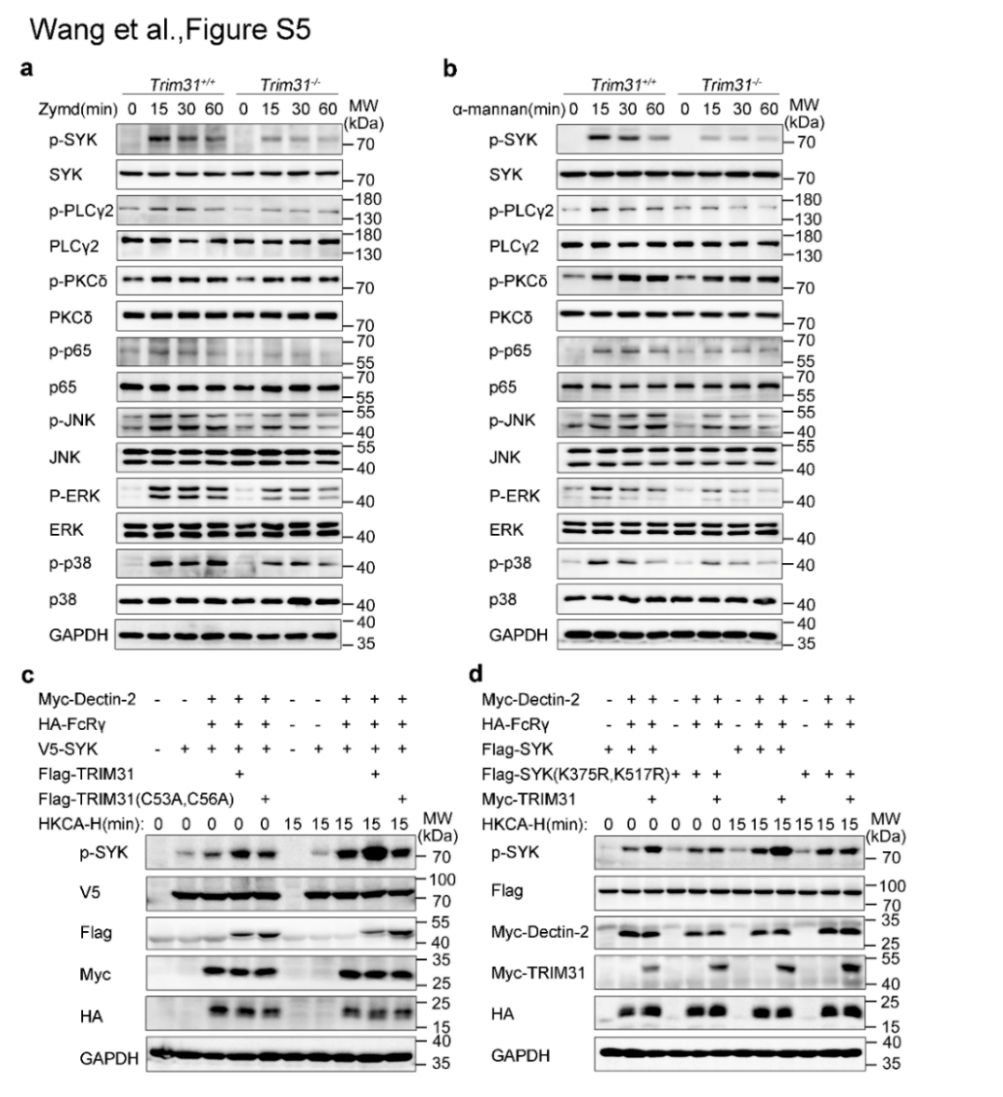
**Figure. S5. TRIM31 promotes SYK phosphorylation.** (**a,b**) Immunoblot analysis of phosphorylated and total proteins in lysates of *Trim31^+/+^* and *Trim31^−/−^* BMDMs stimulated with Zymd (100 µg/ml) (**a**) or α-mannan (100 µg/ml) (**b**), the BMDMs primed by IL-4 (10ng/ml) overnight. (**c**) Immunoblot analysis of SYK phosphorylation and activation in HEK293T cells transfected by various combinations of plasmids expressing V5-SYK, Myc-Dectin-2, HA-FcRγ, Flag-TRIM31 or Flag-TRIM31 (C53A, C56A), cells were unstimulated or stimulated by HKCA-H (MOI, 1) for 15 min. (**d**) Immunoblot analysis of SYK or SYK (K375R, K517R) phosphorylation and activation in HEK293T cells transfected by various combinations of plasmids expressing Flag-SYK, Flag-SYK (K375R, K517R), Myc-Dectin-2, HA-FcRγ and Myc-TRIM31, cells were unstimulated or stimulated by HKCA-H (MOI, 1) for 15 min. WCL respectively probed with antibodies (left margins). All the experiments were repeated at least twice with similar results, and the representative data are shown (**a-d**).


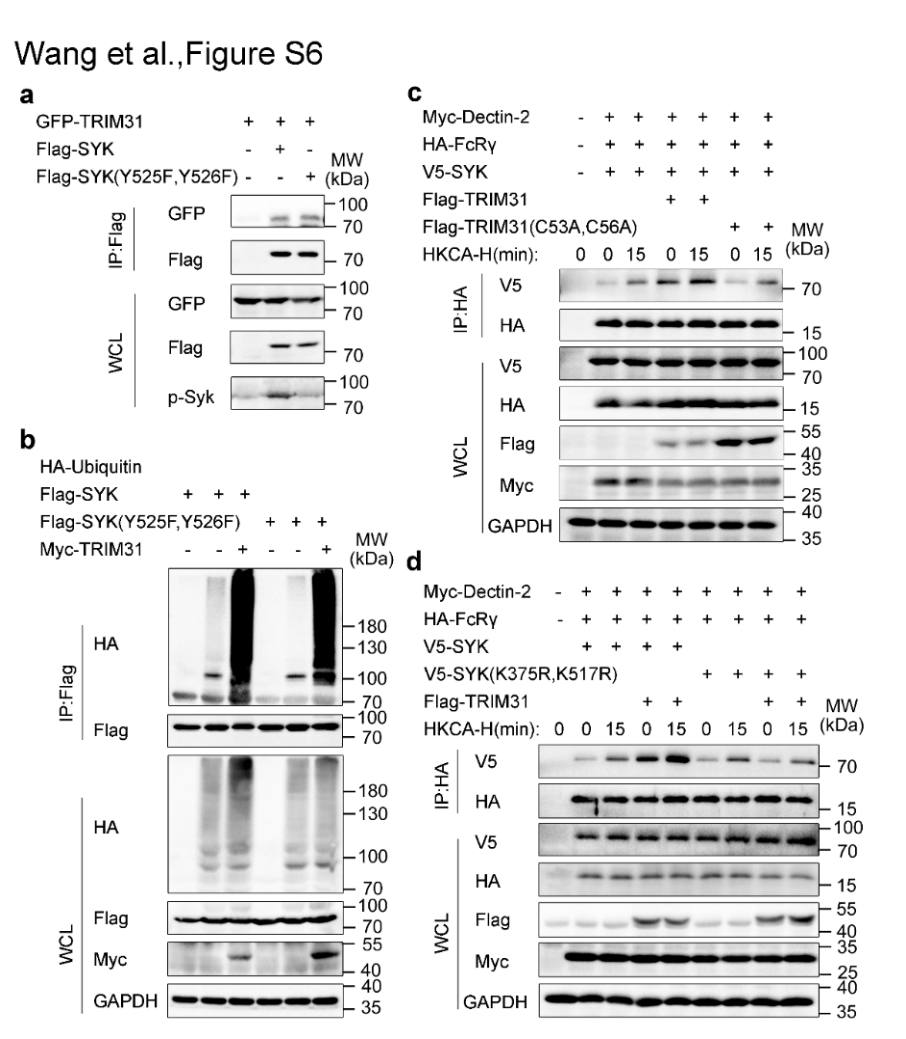
**Figure. S6. TRIM31 promotes the recruitment of SYK to FcRγ.** (**a**) Co-immunoprecipitation analysis of GFP-TRIM31 with Flag-SYK or Flag-SYK (Y525F, Y526F) plasmids transfected into HEK293T cells, followed by IP with anti-Flag, probed with anti-GFP. (**b**) Co-immunoprecipitation analysis of Flag-SYK or Flag-SYK (Y525F, Y526F) ubiquitination in HEK293T cells transfected with Myc-TRIM31 and HA-ubiquitin (WT), followed by IP with anti-Flag, probed with anti-HA. (**c**) HEK293T cells were transfected with Myc-Dectin-2, HA-FcRγ, V5-SYK, Flag-TRIM31 or Flag-TRIM31 (C53A, C56A) by various combinations, cells were unstimulated or stimulated with HKCA-H (MOI, 1) for 15 min. Cell lysates were IP with anti-HA and WCL respectively probed with antibodies (left margins). (**d**) HEK293T cells were transfected with Myc-Dectin-2, HA-FcRγ, V5-SYK or V5-SYK (K375R, K517R) and Flag-TRIM31 by various combinations, cells were unstimulated or stimulated with HKCA-H (MOI, 1) for 15 min. Cell lysates were IP with anti-HA and WCL respectively probed with antibodies (left margins). All the experiments were repeated at least twice with similar results, and the representative data are shown (**a-d**).

**Table S1**

| **Antibody** | **Clone** | **Distributor** |
| --- | --- | --- |
| Anti- Rabbit- phospho-PLCγ2 (Tyr759) | #3874 | Cell signaling Technology |
| Anti- Rabbit- PLCγ2 | #3872; | Cell signaling Technology |
| Anti- Rabbit- phospho-PKCδ (Th505) | #9374; | Cell signaling Technology |
| Anti- Rabbit- PKCδ | #9616; | Cell signaling Technology |
| Anti- Rabbit- phospho-Syk (Tyr525/526) | #2710; | Cell signaling Technology |
| Anti- Rabbit- Syk | #13198; | Cell signaling Technology |
| Anti- Rabbit- phospho-p44/42 MAPK (Erk1/2) (Thr202/Thr204) | #4370; | Cell signaling Technology |
| Anti- Rabbit- p44/42 MAPK (Erk1/2) | #4695; | Cell signaling Technology |
| Anti- Rabbit- phospho-SAPK/ JNK (Thr183/Tyr185) | #4671; | Cell signaling Technology |
| Anti- Rabbit-SAPK/JNK | #9258; | Cell signaling Technology |
| Anti- Rabbit- phospho-NF-κB p65 (Ser536) | #3033; | Cell signaling Technology |
| Anti- Rabbit- NF-κB p65 | #8242; | Cell signaling Technology |
| Anti- Rabbit- phospho-p38 MAPK (Thr180/Tyr182) | #9215; | Cell signaling Technology |
| Anti- Rabbit- p38 MAPK | #8690; | Cell signaling Technology |
| Anti- Rabbit- SHP-2 | #3397; | Cell signaling Technology |
| Anti- Rabbit-SHP-1 | #3759; | Cell signaling Technology |
| Anti- Rabbit- GAPDH | #AB0037; | Abways Technology |
| Anti- Rabbit- FcRγ | #06-727; | Upstate Biotechnology |
| Anti- Mouse- Ub (P4D1) | #sc-8017; | Santa Cruz Biotechnology |
| Anti- Rabbit- Ub (K27) | #ab181537; | Abcam |
| Anti- Rabbit- Ub (K48) | #4289; | Cell signaling Technology |
| Anti- Rabbit- Ub (K63) | #5621; | Cell signaling Technology |
| Anti- Rabbit- TRIM31 | #AV34717; | Sigma-Aldrich |
| Anti- Rabbit- GFP | #2956; | Cell signaling Technology |
| Anti- Mouse- Myc | #TA150121; | Origene |
| Anti- Mouse- HA | #TA180128; | Origene |
| Anti- Mouse- Flag M2 | #F1804; | Sigma-Aldrich |
| Anti- Mouse- Flag M2-Affinity Gel | #A2220; | Sigma-Aldrich |
| Anti- Mouse- V5 | #abs830008 | Absin |
| Anti- Rabbit- V5 | #13202 | Cell signaling Technology |
| Anti- Rabbit- Dectin1 | #SC-73897 | Santa Cruz Biotechnology |
| Anti- Mouse- Dectin1 | #ab82888 | Abcam |
| Anti- Mouse- Syk | #ALX-804-480-C100; | Enzo Life Sciences |
| Anti-Rabbit- IgG | #3900; | Cell signaling Technology |
| FITC anti-mouse CD3 | #100204; | Biolegend |
| PE/Cy7 anti-mouse CD4 Antibody | #100422; | Biolegend |
| PE anti-mouse IFN-γ Antibody | #505808; | Biolegend |
| APC anti-mouse IL-17A Antibody | #506916; | Biolegend |

**Table S2**

| **Gene** | **Forward** | **Reverse** |
| --- | --- | --- |
| pcDNA3.1-Myc-Dectin-2 | CCCAAGCTTGCCGCCACCATGGAGCAGAAGCTGATCTCAGAGGAGGACCTGATGATGCAAGAGCAGCAACCT | CGCGGATCCTCATAGGTAAATCTTATTCATCTCA |
| pcDNA3.1-Myc-Dectin-3 | CCCAAGCTTGCCGCCACCATGGAGCAGAAGCTGATCTCAGAGGAGGACCTGATGGGGCTAGAAAAACCTC | CGCGGATCCCTAGTTCAATGTTGTTCCAGG |
| pcDNA3.1-Myc-Mincle | CCCAAGCTTGCCGCCACCATGGAGCAGAAGCTGATCTCAGAGGAGGACCTGATGAATTCATCTAAATCATCTG | ATTTGCGGCCGCTTAAAGAGATTTTCCTTTGTTC |
| pcDNA3.1-Myc-FcRγ | CCCAAGCTTGCCGCCACCATGGAGCAGAAGCTGATCTCAGAGGAGGACCTGATGATTCCAGCAGTGGTCTTG | CGCGGATCCCTACTGTGGTGGTTTCT |
| pcDNA3.1-Flag-TRIM10 | CCCAAGCTTGCCGCCACCATGGACTACAAGGATGACGATGACAAGATGGCCTCTGCTGCCT | CCGGAATTCTCAGGAGCTCAGGGA |
| pcDNA3.1-Flag-TRIM40 | CCGGAATTCGCCGCCACCATGGACTACAAGGATGACGATGACAAGATCCCTTTGCAGAAGGACAACCAGGAGGAG | CCGCTCGAGTCAGAGCTTCTGAGGGGGCTGAAGAAGCAATTC |
| pcDNA3.1-Flag-TRIM62 | CGGGATCCGCCGCCACCATGGATTACAAGGATGACGACGATAAGGCGTGCAGCCTCAAGGACGAGC | CGGAATTCCTAGATGCGGACGGTGTTGATCCGC |
| pCMV-N-Myc -PKCδ | CGCGGATCCATGAGGGAAAGGCAGC | CCGGAATTCCCTCAATCTTCCAGG |
| pcDNA3.1-Myc-SHP-2 | CCCAAGCTTGCCGCCACCATGGAGCAGAAGCTGATCTCAGAGGAGGACCTGATGACATCGCGGAGATGGTT | CGCGGATCCTCATCTGAAACTTTTCTGCTGT |
| pcDNA3.1-V5-SHP-1 | CCCAAGCTTGCCGCCACCATGGGTAAGCCTATCCCTAACCTCTCCTCGGTCTCGATTCTACGATGGTGAGGTGGTTTC | CCGGAATTCTCACTTCCTCTTGAGGGA |
| pcDNA3.1-Myc-TRIM31 | CCCAAGCTTGCCGCCACCATGGAGCAGAAGCTGATCTCAGAGGAGGACCTGATGGCCAGTGGGCAG | CCGGAATTCTTAGCTTGAAGGAACCTC |
| pcDNA3.1-Myc-CARD9 | CCCAAGCTTGCCGCCACCATGGAGCAGAAGCTGATCTCAGAGGAGGACCTG ATGTCGGACTACGAGA | CGCGGATCCCTAGGAGCCCTCAGT |
| pcDNA3.1-Flag-BCL10 | CCCAAGCTTGCCGCCACCATGGACTACAAGGATGACGATGACAAGATGGAGCCCACCGCAC | CGCGGATCCTCATTGTCGTGAAACAGTACGTGA |
| pcDNA3.1-Flag-SYK | CCCAAGCTTGCCGCCACCATGGACTACAAGGATGACGATGACAAGATGGCCAGCAGCGGCAT | CGCGGATCCTTAGTTCACCACGTCATAG |
| pcDNA3.1-V5-SYK | CCCAAGCTTGCCGCCACCATGGGTAAGCCTATCCCTAACCCTCTCCTCGGTCTCGATTCTACGATGGCCAGCAGCGGCAT | CGCGGATCCTTAGTTCACCACGTCATAG |
| pcDNA3.1-Myc-SYK | CCCAAGCTTGCCGCCACCATGGAGCAGAAGCTGATCTCAGAGGAGGACCTGATGGCCAGCAGCGGCAT | CGCGGATCCTTAGTTCACCACGTCATAG |
| pcDNA3.1-Myc-SYK  (1-262) | CCCAAGCTTGCCGCCACCATGGAGCAGAAGCTGATCTCAGAGGAGGACCTGATGGCCAGCAGCGGCAT | CGCGGATCCTTAGTTCACCACGTCATAG |
| pcDNA3.1-Myc-SYK  (117-635) | CCCAAGCTTGCCGCCACCATGGAGCAGAAGCTGATCTCAGAGGAGGACCTGACTGGGCCCTTTGAGGATTTG | CGCGGATCCTTAGTTCACCACGTCATAG |
| pcDNA3.1-Myc-SYK  (371-635) | CCCAAGCTTGCCGCCACCATGGAGCAGAAGCTGATCTCAGAGGAGGACCTGACGCTGGAAGACAAAGAACTGG | CGCGGATCCTTAGTTCACCACGTCATAG |
| pcDNA3.1-Flag-SYK(Y525/526F) | AACTTCTTCAAGGCCCAGACCCAT | TTCATCAGCACGCAGTGCTTT |
| pcDNA3.1-Flag-SYK(K124R) | TTGCGGGAAAACCTCATCAGG | ATCCTCAAAGGGCCCAGTCTT |
| pcDNA3.1-Flag-SYK(K165R) | GAAAGGATGCCTTGGTTCCAT | ATGGGCTGTGGTAGCGATCAG |
| pcDNA3.1-Flag-SYK(K261R) | CAAAGGATCGGCACACAGGGA | ACATGGGACAGTAAGAACTCT |
| pcDNA3.1-Flag-SYK(K368R) | CGACGGCTGCTGACGCTGGAA | GTCCAGGTAAACCTCCTTGGG |
| pcDNA3.1-Flag-SYK(K375R) | GACAGGGAACTGGGCTCTGGT | TTCCAGCGTCAGCAGCTTTCG |
| pcDNA3.1-Flag-SYK(K386R) | GTGAGGAAGGGCTACTACCAA | AGTTCCAAAATTACCAGAGCC |
| pcDNA3.1-Flag- SYK(K517R) | TCCAGGGCACTGCGTGCTGAT | AAGTCCGAAATCACTGATCTT |

**Table S3**

| **Genes** | **Forward sequence** | **Reverse sequence** |
| --- | --- | --- |
| Mouse *Act* | CCACACCCGCCACCAGTTCG | TACAGCCCGGGGAGCATCGT |
| Mouse *Il17a* | GGAGAGCTTCATCTGTGTCTCTG | TTGGCCTCAGTGTTTGGACA |
| Mouse *Il17f* | AGACAGCACCATGAACTCCG | ACAATGGGCTTGACACAGGT |
| Mouse *Ifng* | TCAAGTGGCATAGATGTGGAAGAA | TGGCTCTGCAGGATTTTCATG |
| Mouse *Trim31* | GGCCTTGGATTTCTGTACTTTCACATC | TGGGCCTGAACGTATTCTTATTCACAG |
| Mouse *Syk* | GCCCGTTCTGTGCCTACTGG | TAGCTAACCAAACCCACGGC |

**Table S4**

| **Chemicals** | **Source** | **Code** |
| --- | --- | --- |
| Zymosan Depleted | Invivogen | tlrl-zyd |
| TDB | Invivogen | tlrl-tdb |
| α-mannan | Sigma-Aldrich | 9036-88-8 |
| ATP | Sigma-Aldrich | A7699 |
| Tamoxifen | Sigma-Aldrich | T5648 |
| Corn oil | MedChemExpress | HY-Y1888 |
| IL-4 | R&D Systems | 404-ML-100 |
| GM-CSF | R&D Systems | 415-ML-050 |
| Cell Activation Cocktail (with Brefeldin A) | Biolegend | 423303 |
| Neomin sulfate | Solarbio | N8090 |
| Polymyxin B Sulfate | Solarbio | P8350 |
| Lipofectamine™ 2000 | Invitrogen | 11668019 |
| Lipofectamine™ 3000 | Invitrogen | L3000015 |
| RPMI1640 | Hyclone | SH30809.01B |
| Fetal Bovine Serum | Corning | 35-081-CV |
| 0.25% Trypsin | Gibco | 25200072 |
| Penicillin-Streptomycin (10,000 U/mL) | Gibco | 15140122 |
| Opti-MEM™ | Hyclone | 31985088 |
| DMEM | Hyclone | SH30022.01 |
| PrimeScript RT-PCR Kit | Takara | RR037A |
| SYBR Green PCR Master Mix | Roche | 04887352001 |
| Phosphatase inhibitor | Roche | 04906837001 |
| Protease inhibitor | Sigma-Aldrich | S8820 |
| DAPI | Abcam | ab104139 |
